# Assessing Global Marine Biodiversity Status within a Coupled Socio-Ecological Perspective

**DOI:** 10.1371/journal.pone.0060284

**Published:** 2013-04-11

**Authors:** Elizabeth R. Selig, Catherine Longo, Benjamin S. Halpern, Benjamin D. Best, Darren Hardy, Cristiane T. Elfes, Courtney Scarborough, Kristin M. Kleisner, Steven K. Katona

**Affiliations:** 1 Betty and Gordon Moore Center for Ecosystem Science and Economics, Conservation International, Arlington, Virginia, United States of America; 2 National Center for Ecological Analysis and Synthesis, Santa Barbara, California, United States of America; 3 Center for Marine Assessment and Planning, University of California Santa Barbara, Santa Barbara, California, United States of America; 4 Nicholas School of the Environment, Duke University, Durham, North Carolina, United States of America; 5 Department of Ecology, Evolution and Marine Biology, University of California Santa Barbara, Santa Barbara, California, United States of America; 6 IUCN Global Species Programme/Conservation International, Biodiversity Assessment Unit, Arlington, Virginia, United States of America; 7 *Sea Around Us Project*, Fisheries Centre, University of British Columbia, Vancouver, British Columbia, Canada; McGill University, Canada

## Abstract

People value the existence of a variety of marine species and habitats, many of which are negatively impacted by human activities. The Convention on Biological Diversity and other international and national policy agreements have set broad goals for reducing the rate of biodiversity loss. However, efforts to conserve biodiversity cannot be effective without comprehensive metrics both to assess progress towards meeting conservation goals and to account for measures that reduce pressures so that positive actions are encouraged. We developed an index based on a global assessment of the condition of marine biodiversity using publically available data to estimate the condition of species and habitats within 151 coastal countries. Our assessment also included data on social and ecological pressures on biodiversity as well as variables that indicate whether good governance is in place to reduce them. Thus, our index is a social as well as ecological measure of the current and likely future status of biodiversity. As part of our analyses, we set explicit reference points or targets that provide benchmarks for success and allow for comparative assessment of current conditions. Overall country-level scores ranged from 43 to 95 on a scale of 1 to 100, but countries that scored high for species did not necessarily score high for habitats. Although most current status scores were relatively high, likely future status scores for biodiversity were much lower in most countries due to negative trends for both species and habitats. We also found a strong positive relationship between the Human Development Index and resilience measures that could promote greater sustainability by reducing pressures. This relationship suggests that many developing countries lack effective governance, further jeopardizing their ability to maintain species and habitats in the future.

## Introduction

People appreciate the variety of species in the oceans for their beauty and uniqueness, the natural systems that they collectively create, and the ecosystem services that they support [1]–[3]. Even when there is no direct use for them, species and the ecosystems they help build are widely valued for their existence [2], [4]. Because species have aesthetic, spiritual, educational, and scientific value [5], their loss can generate an enormous amount of public concern, particularly if they have wide public appeal. Biodiversity declines have already motivated the dedication of substantial resources toward protecting and restoring species [6], [7]. Globally, the Strategic Plan for the Convention on Biological Diversity (CBD) recognizes these values and has established the Aichi Biodiversity Targets for signatory countries to achieve by 2020 [8], including Targets 5 and 12, which aim to reduce natural habitat loss by 50% and prevent the extinction of threatened species respectively. Complementary efforts include the 1973 Convention on International Trade in Endangered Species (CITES), U.S. Endangered Species Act of 1973 (ESA; 7 U.S.C. § 136, 16 U.S.C. § 1531 et seq.), Province of Ontario, Canada, Endangered Species Act of 2007 (S.O. 2007 c6) and many others. However, efforts to conserve biodiversity cannot be effective without a framework to assess current extinction risk, level of pressures, and governance factors that can measure progress towards meeting conservation goals. We developed a global assessment of the condition of marine biodiversity using publicly available data to estimate how countries are doing not only in preventing marine species extinctions, but also preserving the natural marine habitats on which many species depend.

Several indices have been valuable for tracking how marine biodiversity is faring at various spatial, temporal, and taxonomic resolutions [9]. Although species diversity indices and assessments have historically focused on terrestrial ecosystems, efforts to quantify and assess marine biodiversity have increased in the last decade [10], [11]. The Living Planet Index is one of the longest running indices of biodiversity, but due to data constraints, tracks a relatively small and taxonomically narrow set of marine populations, although new taxa are added with every iteration [12], [13]. The International Union for the Conservation of Nature's (IUCN) Red List [14] uses a series of internationally accepted criteria to measure which species are most at risk of extinction and how their status is changing through time [15], [16]. Red List assessments for terrestrial species began in the 1960 s; though some marine species were assessed in the 1990 s, the Global Marine Species Assessment (GMSA, http://sci.odu.edu/gmsa) substantially increased that effort beginning in 2005. Although different indices vary in their metrics, they all have an implicit target of at least no additional decline in species and habitats. The assessment we describe adds value and novelty by evaluating both species and key habitats, setting a target reference point for biodiversity beyond ‘no additional loss’, integrating measures of social and ecological pressures that reduce biodiversity, and accounting for social and governance factors that should improve it.

This biodiversity assessment was developed as one of the ten goals that comprise the Ocean Health Index [17], with the objective of measuring how successfully the species and habitats that support biodiversity are being conserved. We track both species and habitats in part due to limited data on marine species status. However, we also explicitly evaluate habitats because of the additional public values associated with maintaining a diverse set of marine environments. In addition, habitat condition can serve as a proxy indicator of species status for species that depend on habitat structure, but are not assessed. More than 90% of marine species have not yet been described [18] and even fewer have been formally assessed for their status. Species and habitat data each have gaps and shortcomings. By using data on both species and habitats, we created an integrated, complementary measure of biodiversity that makes the best use of available data.

Here we explore in greater detail the nature and implications of the biodiversity results presented in the global Ocean Health Index [17], focusing in particular on country-by-country results. Results from our analyses reveal geographic, political, and governance patterns that may help explain successes and failures in biodiversity conservation. We also assess how specific habitat types and taxonomic groups affect Index scores. Finally, we highlight where strategic action is likely to best promote biodiversity, where key data gaps remain, and how different assumptions and values affect our assessment and understanding of the current and future condition of biodiversity.

## Methods

### Ocean Health Index biodiversity framework

The general methods for calculating the biodiversity scores are provided in Halpern *et al.* [17]; here we briefly summarize them and explain additional analyses that were conducted. Scores for overall biodiversity, species, and habitats were a combination of a current state (status relative to a reference point) and a likely future state (∼5 years in the future), estimated by using data on trend, pressures and resilience (Table S1–S2).

For species, our target reference point was for all species to be categorized by the IUCN Red List Criteria [14] as Least Concern. A taxon is assessed to be at Least Concern when it does meet the criteria for listing as Critically Endangered, Endangered, Vulnerable or Near Threatened. Least Concern taxa are usually widespread and abundant, with a small likelihood of extinction at present [14]. This target was meant to reflect the societal goal of species preservation [19]. For habitats, our reference point was the extent or condition of the habitats in the 1980 s, a temporal reference point that was chosen as an achievable, yet ambitious target [19]. One factor in setting the reference point to this time period was the limited data available from earlier time periods. Since changes in species and habitat status may not manifest themselves immediately and because there were data gaps in the existing information, we used the whole time series available to estimate the near-future (∼5 yr) trend of species and habitats (Text S1).

Pressures were defined as anthropogenic stressors that negatively affect species or habitats. We included 15 stressors that fell into five broad categories: fishing pressure, habitat destruction, climate change, water pollution, and species introductions (Table S1). We also included a measure of social pressures based on indicators of governance in each country. Each pressure was weighted according to the expected severity of its impact on the status indicator (Table S1). For example, corals are more sensitive to increases in ocean temperature than to alien species, so changes in ocean temperature were weighted more heavily than alien species. Pressures belonging to a given category were assessed cumulatively.

The total score was based on an equal weighting of the current status and its likely future state. Likely future state was a function of trends, pressures and resilience and was defined as follows:
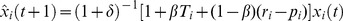
(1)Where *r*_i_ is resilience and *p*_i_ is pressures, which were scaled such that 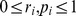, with 1 being the maximum value in both cases. The trend (*T_i_*) was constrained to −1.0 ≤ *T_i_* ≤ 1.0. A discount rate (*δ*) was included in the equation, but was approximated to be 0, because the likely future state is an assessment in the very near future [17]. Beta (*β*) represented the relative importance of the trend versus the resilience and pressure terms in determining the likely trajectory of the goal status into the near future. We assumed β = 0.67 based on the idea that in the absence of significant changes in human actions, recent trends are likely to continue into the near (∼5 yr) future and the direct measure of trend is a better indicator of the near-term direction and magnitude of change than the indirect measures of pressure and resilience [17]. Pressures were a function of both ecological and social pressures (Table S1), which were weighted equally [17]. For each habitat and for the species sub-goal, we ranked pressures by the sensitivity of the habitat or the suite of species we analyzed to that pressure as ‘high’ (score  = 3), ‘medium’ (score  = 2) or ‘low’ (score  = 1) (Table S1). The ranks were used to weight the relative contribution of each of the pressure categories to the overall pressure score. To calculate resilience, we included measures of ecological integrity (*Y_E_*), regulations aimed at addressing ecological pressures (*G*), and social integrity (*Y_S_*) (Table S2). The first two measures address ecological resilience while the third addresses social resilience. Resilience is then calculated as:
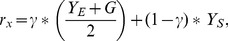
(2)where the three types of measures are all scaled 0-1, and γ = 0.5 so that ecological and social resilience measures were equivalent [17]. The mean across the 6 Worldwide Governance Indicators (WGI) value was used to measure social resilience and 1-WGI was used to indicate social pressures.

### Species

To calculate species status, we used data from IUCN Red List Assessments of extinction risk for 2285 marine species (Table S3) [20]–[25] to calculate average extinction risk of the species within an EEZ region. Extinction risk was based on the application of IUCN Red List Categories and Criteria [14]. Global Red List assessments quantify extinction risk across the entire species range. Weights (*w_i_*) were assigned based on the extinction risk of each *i* species, following the previously determined weighting scheme developed by Butchart *et al.* [16]. The weights were assigned as equal increments across extinction risk categories (Extinct, Critically Endangered, Endangered, Vulnerable, Near Threatened and Least Concern; Table S4). Our analyses excluded all species that were assessed as Data Deficient (N = 633), a category which may contain species that are threatened, but for which insufficient data are currently available to quantify their risk of extinction. As such our results may be optimistic if many of these species are at higher risk of extinction.

We scaled the lower end of status to correspond with a 75% extinction loss. Studies suggest that 75% extinction is comparable to the five mass extinctions documented during geological history [26] and would constitute a catastrophic loss of biodiversity. With this scaling, a score of 0 would be achieved if all species were Critically Endangered. The species status score 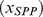 was calculated as the area-weighted average of the IUCN Red List status for all species within each EEZ:
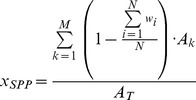
(3)For each 0.5 degree grid cell (*k*) within an EEZ, the risk status weight (*w*) for each species (*i*) present was summed and divided by the total number of assessed species present in the cell (*N*). This average species risk was subtracted from 1 so that the presence of more threatened species produced a lower score. The result was then weighted by the ocean area (*A_k_*) occupied by the cell, relative to the total area of the assessment region (*A_T_*), where *M* was the number of grid cells in the assessment region.

We calculated trend as the average of the population trend assessments provided in the Red List species assessment for all species within a region, assigning values of 0.5, 0 and −0.5 for increasing, stable and decreasing trends, respectively. Trend information was available for 48% of species.

Pressures to species included all ecological pressures assessed in the general framework (Table S1) [17], except human pathogens. All social pressures derived from the World Bank's Worldwide Governance Indicators (WGI) were also included (Table S1). Resilience measures included regulations promoting ecological resilience that address identified pressures as well as social resilience metrics from the WGI (Table S2).

### Habitats

Habitat status was based on publicly available global data for the following: mangroves [27], coral reefs [28], [29], seagrass beds [30], salt marshes [31]–[35], sea ice [36], and subtidal soft-bottom [37]. Habitat status 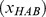
was assessed as the average of the condition estimates (*C*) for each *k* habitat present in a region, such that:
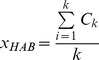
(4)where *C_k_*  =  *C_c_/C_r_* and *C_c_* was defined as the current condition and *C_r_* was the reference condition specific to each *k* habitat present in the region. Each country was assessed only for those habitats that were native and present in the reference years. For example, Canada was not assessed on the status of non-existent tropical coral reefs or mangroves and the Netherlands was not assessed on historical salt marsh extent from 100 years ago. Data from the 1980 s were used when possible to estimate a reference condition; if unavailable, data in the year range of 1975–1995 were used instead. Current years were considered to be 2001–2010. For some habitats, gap-filling was necessary (Text S1). Specific methods for calculating trends varied by habitat type, but we generally used the entire available time period of data from the present to the 1980 s to fit linear regressions to calculate the change in habitat extent or condition (Text S1) because time series data for habitats were often quite sparse. Using the whole time period available allowed us to make better use of periods of intense sampling, while smoothing annual variability.

Ecological pressures varied by habitat and were applied per-country depending on which habitats were present in each country (Table S1). Social pressures were assumed to affect all habitats equally and were estimated using the WGI scores. Similar to the species scores, we used 1-WGI as a measure of social pressures and WGI as a measure of social resilience. Governance metrics varied by habitat, depending on which pressures were ranked highly for each habitat (Table S2). Social resilience was assumed to be equally relevant to all habitats and all countries.

### Statistical analyses

We conducted several correlation analyses to further understand patterns and drivers of our results. We examined the correlation between habitats and species scores to determine whether or not scores between the two were related. Then we analyzed how current status scores related to likely future state scores, which enabled us to identify whether countries that scored high on status generally scored higher in the near-term future. We also examined the relationship between the Human Development Index and resilience scores to determine whether resilience measures were related to the degree of development.

### Species sensitivity analyses

Species results can be affected by inherent biases in the selection of taxa that have been assessed for extinction risk. To determine how well the species in our analysis represented overall diversity, we compared the number of species in our analysis to all catalogued, mapped and assessed species at a coarse taxonomic resolution (Cnidaria, Mammalia, Pisces, Reptilia, Plantae, and Other; Text S1). Then we grouped all species following the finer taxonomic grouping used by the IUCN assessment process (Table S3), which typically convenes experts to conduct comprehensive species assessments by taxonomic group. With this finer taxonomic resolution, we were able to consider both taxonomic and spatial biases. Spatial biases were determined by qualitative examination of where species have been assessed relative to the diversity present in those locations using spatially explicit 0.5 degree resolution data [38]. Taxonomic biases were analyzed using a jackknife approach where we excluded each of the 15 distinct taxonomic groups to examine that group's effect on the score for each EEZ. We were also able to evaluate geographic patterns in the location of species assessments relative to the spatial extent of mapped biodiversity for these species. To determine whether species richness had an effect on average extinction risk, we used a Pearson's correlation analysis.

**Figure 1:**
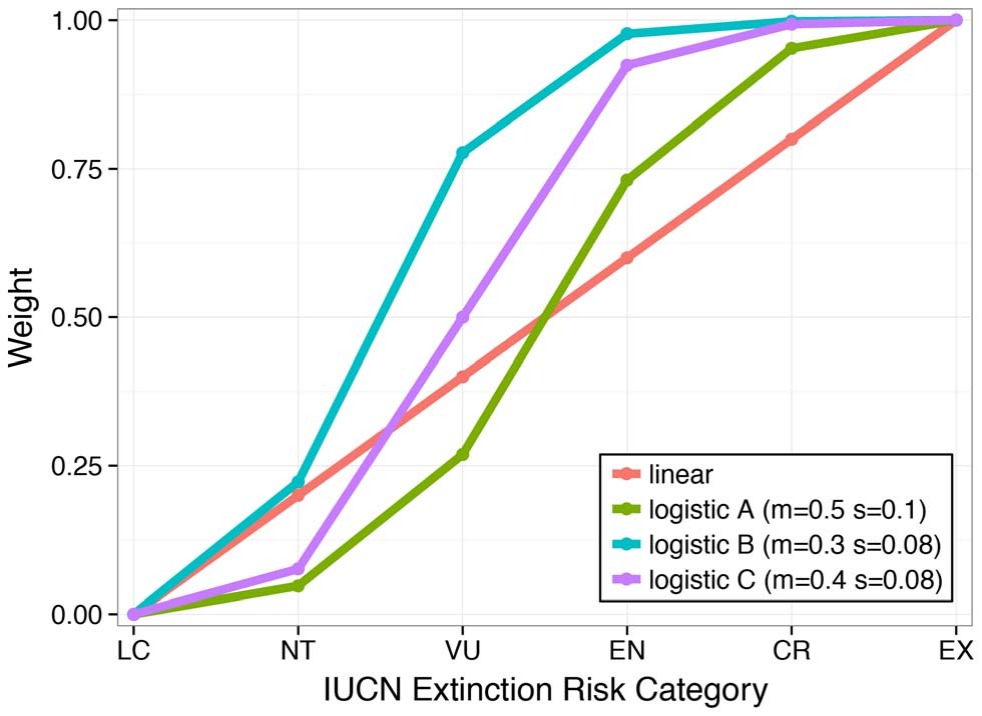
Weighting schemes used to explore how the weights applied to IUCN categories affect species scores. In the main analysis, a linear weighting scheme (red line) was applied. Three different logistic weighting schemes (as defined by the function 1/(1+*e* ^−x−m/s^) were also explored by varying the location (*m*) and scale (*s*) parameters. The endpoints were defined for all schemes (LC = 0, EX = 1). The goal of these additional weighting schemes was to compare them to linear weights to explore how the ways that people value or are aware of species loss or extinction risk affects scores.



**Figure 2:**
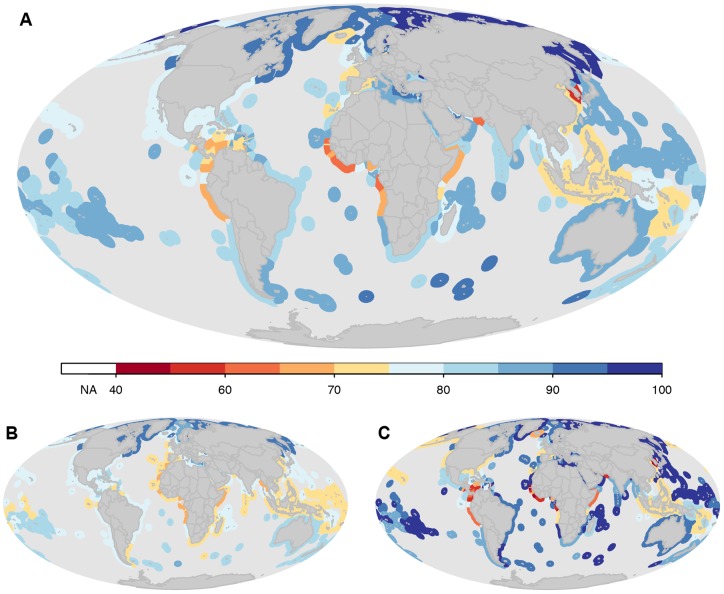
Scores for (A) overall biodiversity, (B) species, and (C) habitats.

**Figure 3:**
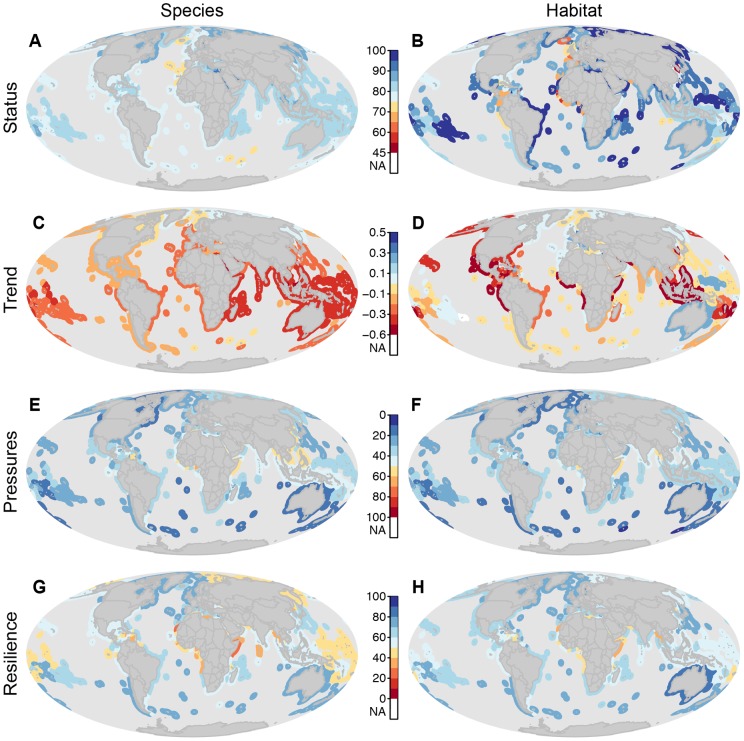
Scores for species status (A), habitat status (B), species trend (C), habitat trend (D), species pressures (E), habitat pressures (F), species resilience (G), and habitat resilience (H). Note changes in color ramps for the different panels.

We also explored the potential effect of nonlinear weighting schemes for the different IUCN extinction risk categories. Specifically we used three different logistic functions (Fig. 1) to approximate different ways people may value the change in risk status for species: 1) ‘high interest’, where the initial shift of species from Near Threatened into Vulnerable is perceived as bad and thus given disproportionate weight (logistic B; Fig. 1), 2) ‘moderate interest’, where there is less concern about species that are Near Threatened but greater interest once species shift to Vulnerable and higher risk categories (logistic C; Fig. 1), and 3) ‘low interest’, where there is relatively little interest until species are Endangered or worse (logistic A; Fig. 1). We used the following logistic equation to describe all three curves, modifying the location (*m*) and scale (*s*) parameters to change the shape.

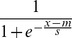
(5)Although this equation constrained the possible shape of the curve, it allowed for a consistent and transparent means to define the logistic curve.

Finally, we also tested the effect of changing the lower-bound reference point. Although the lower bound of 75% loss of species has empirical support [26], people may consider ‘catastrophic loss’ of species as anything from 50% of all species to 100% of all species. We therefore recalculated the species scores using a range of values from 50–100% for this lower-bound reference point.

### Habitat sensitivity analyses

To determine which habitats had the greatest effect on the scores, we explored the individual status and trends scores for each habitat. Then we analyzed the correlation between individual habitat scores and overall habitat scores. Because not all habitat combinations are found at all latitudes, linear regressions were obtained separately for three broad latitudinal regions (tropical: −30° to +30°; temperate: −30° to −60° and +30° to +60°; and polar: >60°, <−60°). Habitats were excluded when they occurred in less than five EEZs within that latitudinal range (Table S6).

## Results and Discussion

**Figure 4:**
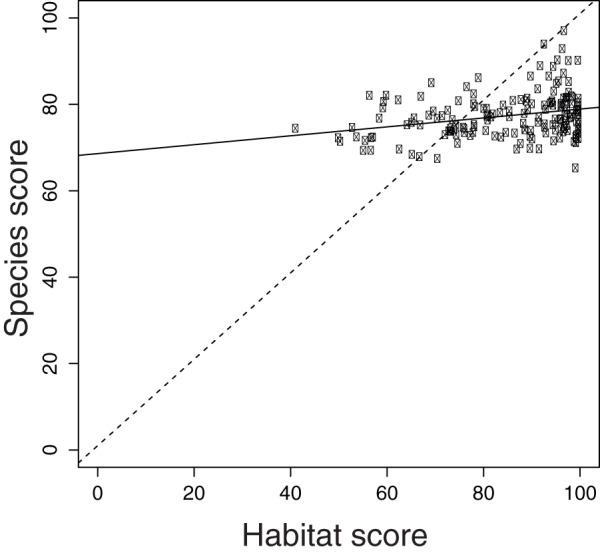
Relationship between habitat and species scores for all EEZ regions. Dashed line represents a 1:1 relationship. Solid line is the regression line. (R-squared  = 0.07; p-value  = 0.0004).

**Figure 5:**
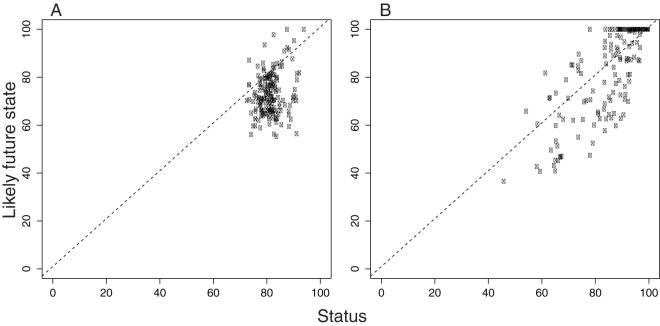
Relationship between current status and likely future state for A) species and B) habitats for all EEZ regions. Dashed line represents a 1:1 positive relationship, with values below this line indicating where the likely future state may be worse than the current state.

Previous studies have shown that many marine species and habitats are declining [30], [39]–[41]. Here we provide an integrative picture of how species and the key habitats that support them are faring for each country's EEZs (Figs. 2–3). Scores varied by country (Table S6; Figs. 2–3), and countries that scored high for species did not necessarily score high for habitats (Fig. 4). Likely future state results suggest a more negative picture for biodiversity in the near-future (Fig. 5), driven mostly by negative trends for species and habitats (Fig. 3C, 3D). For species, nearly all countries had negative likely future states, suggesting that most marine species will probably continue to decline.

**Figure 6:**
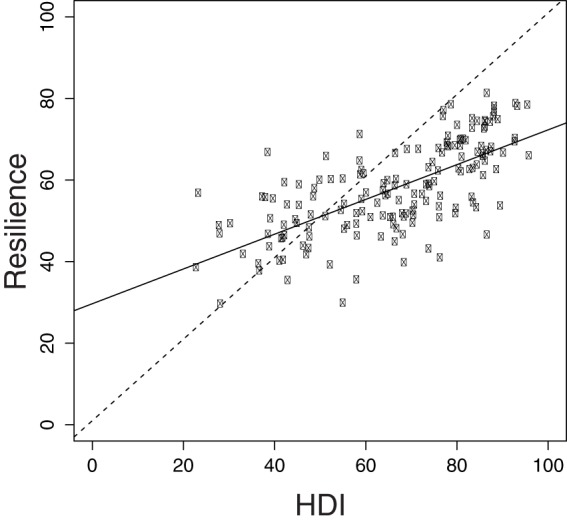
Relationship between Human Development Index (HDI) and resilience scores. For the HDI, raw scores were multiplied by 100 to facilitate comparison. Solid lines are the regression lines (HDI vs. Resilience: R-squared  = 0.46; p-value <0.0001). Dashed line is the 1:1 positive relationship.

The area-weighted mean score for overall biodiversity for all EEZs was 83. Generally, per country scores were lower for species (area-weighted mean  = 79) than for habitats (area-weighted mean  = 88). Biodiversity scores were lowest in the tropics, particularly in the EEZs of West African countries, although Togo, Benin, and Sao Tome and Principe scored relatively higher (Table S6; Fig. 2). Lower scores in many West African countries may be due in part to poor fisheries management and widespread illegal fishing [42]. Unsustainable fishing levels or methods can have negative impacts on biodiversity. In addition, most West African countries scored in the lowest 25% of all countries in terms of government effectiveness indicators based on the Worldwide Governance Indicators (WGI) so their resilience scores were low. South Korea and several countries in Latin America, including Colombia, Peru, Nicaragua, and Grenada, also had relatively low biodiversity scores (Table S6, Fig. 2). Countries that scored the highest included Finland, Russia, and Canada. Although some countries that received high scores also ranked high on the Human Development Index (HDI), there was no significant correlation between the degree of development and overall biodiversity scores. However, there was a strong positive relationship between HDI and resilience scores, which suggests that many less developed countries may lack effective governance measures to maintain biodiversity (Fig. 6; R-squared  = 0.46; p-value <0.0001).

Generally, species scores were lower than habitat scores, but were also less variable (Fig. 2). West African countries scored the lowest, while Somalia and Myanmar also scored relatively low, in part because they scored poorly on resilience and had among the highest pressures scores (Table S6; Figs. 3E, 3G). The highest species scores were found in the EEZs of Finland, Canada, Cyprus, Denmark, and Russia, countries that are more developed. However, other developed countries such as Ireland, Spain and Portugal had lower scores, suggesting that there is not always a clear relationship between development and species scores. In spite of relatively high scores for current species status, negative trends indicate that in nearly every country in the world species are expected to decline (Table S6; Figs. 3A, 3C). Consequently, the likely future state of the vast majority of countries was considerably worse than their current status for species (Fig. 5A). Likely future state scores for species were, on average, nearly 9 points less than current status scores.

Our results are consistent with previous work that has found that pressures on marine species are highest in Southeast Asia [37], [43], but the relative proportion of species that are classified as threatened based on IUCN Red List Categories and Criteria for these regions is similar to that for other geographic areas (Fig. S4). Indeed, we found no evidence of a relationship between species richness and average area-weighted extinction risk (Pearson's correlation  = 0.0677). Although there were higher numbers of species at risk in the tropics, the relative risk of species extinctions across regions was relatively homogenous (Fig. 2B). This result may be due in part to our use of global species assessments, which may fail to identify areas where extinction risk is higher at more local scales. Our use of a relative risk metric across many taxa may also have resulted in less geographic variability. By evaluating species loss in relative terms, we assume that the loss of a species in a location with 300 species may matter more than it does in a region that has 3000 native species. However, people may care more about the absolute number of species at risk, or exactly which species are at risk, an issue we explored when setting reference points and which we discuss further below.

Several sampling and taxonomic biases that are inherent in any effort to assess the condition of overall biodiversity from a subsample of species may have affected our results. For example, given the high number of species in the tropics, there may be a greater likelihood of failing to identify an at-risk species, leading overall species risk to be underestimated. Our results may also have been affected by the relatively small subset of total species [18] that have been assessed (Table S5; Fig. S1). Although most of the species assessed were tropical (Fig. S2), when we compared the ratio of species assessed to species mapped (Fig. S3), we found no consistent geographic bias. In addition, when we analyzed how sensitive overall results were to the removal of each taxonomic group, we were able to show that our results were robust (Table S7; Figs. S5, S6).

The relatively narrow range of species scores can be attributed to several factors. Because all species risk values were weighted equally and averaged, known taxa-specific threat patterns [20], [21], [23]-[25] may be less pronounced. Different results may have been expected had we averaged across taxonomic groups instead of species. By averaging across taxonomic groups, the mean extinction risk of taxonomic groups represented by few species (e.g. reptiles) would influence the score just as much as those with many species (e.g. corals). Using taxonomic groups as the unit for averaging may be more appropriate for an assessment of functional diversity, but for the purposes of this study where we were focusing on existence value it was deemed more appropriate to consider all species equally. We used a weighting scheme based on Butchart *et al*. [15], which uses ‘equal-step’ increments to weight each category of increasing threat. Under this weighting, an area with three Vulnerable species has the same average value as an area with a single Critically Endangered species (Table S4). This approach causes results to be driven not only by a few Critically Endangered species, but also by relatively large numbers of species that are at lower risk [15]. We chose this approach because we were concerned with the existence value of all species and therefore species at lower levels of extinction risk were also important to consider. Nevertheless, the weighting scheme still ensures that lower risk species influence scores less than Critically Endangered species.

Ultimately, weights should be driven by the societal value given to preventing species loss, so other weighting schemes may also be appropriate. If public response to species losses is averaged across people with different values and thresholds of sensitivity, it may behave as a multi-function response curve. In other words, even though individual values linked to species loss may not be linear, when considered in aggregate, they may approach a linear function. For example, Zavaleta *et al*. found that the relationship between species loss and reduction in ecosystem function across multiple functions appears to be linear even though individual functional relationships may vary [44].

**Figure 7:**
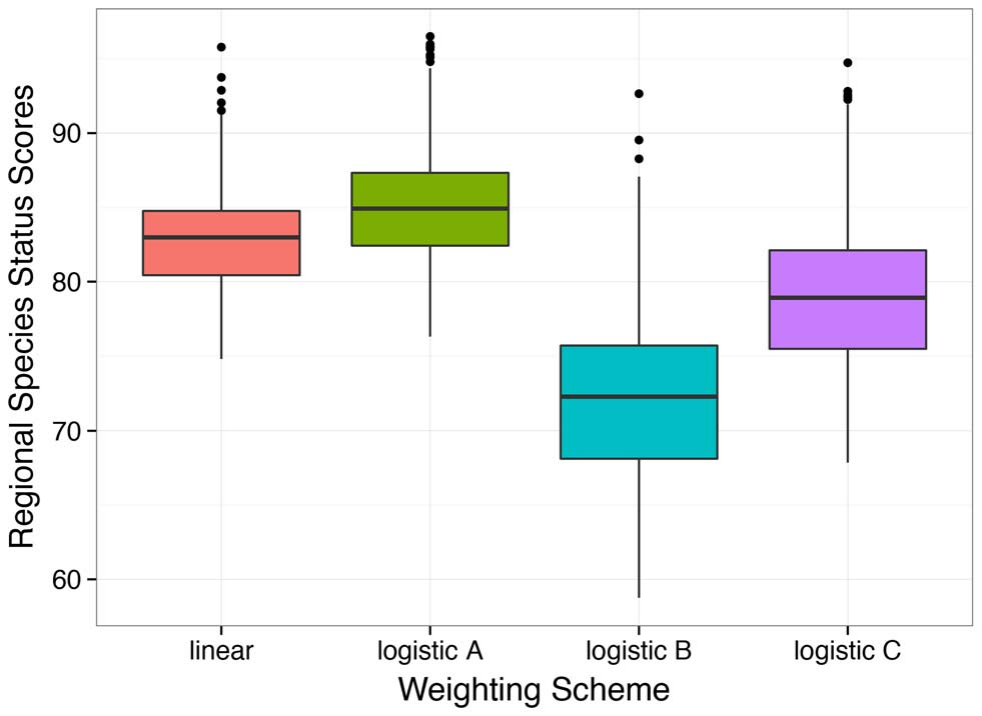
Box plot of EEZ regional species status scores by weighting scheme. Parameters for weighting schemes are in Fig. 1. The median value divides the box, which extends from the first (25%) to the third (75%) quartiles of the distribution. Whiskers extend to the last point within this interquartile range * 1.5 and dots indicate outliers.

However, many other value relationships may exist and likely vary according to location, species identity or richness [45], [46], and cultural context. To determine the effect of weighting on species scores, we explored a series of logistic weighting schemes in addition to the linear model we used for species score calculations (Fig. 1). Public concern as a function of species loss may follow a general logistic pattern, with different possible shapes depending on people's awareness or the value they place on species risk or loss. For example, people may recognize species loss only when a valued species like a predator that keeps a “pest” species under control goes extinct or when many species have been lost, whereas others may care more about the first species that is lost or becomes Critically Endangered. Whether the public response is driven by the identity, initiation or cumulative effect of species loss, each of these situations can be approximated as a logistic relationship between public value and species extinction risk [45]. Logistic weighting schemes that weight the shift from Near Threatened to Vulnerable more heavily than in a linear model (i.e., ‘high interest’ and ‘moderate interest’ scenarios) can lower the mean species score (Figs. 7, S7), partly because of the number of species in each of these categories relative to each other and to other risk categories (Fig. S8). In our analyses, 31% of assessed species were in these two categories, roughly split equally between them (Fig. S8). Therefore the weights applied to these two categories greatly influenced the species scores. Thus, if the ‘high interest’ scenario better captures how most people feel, then our scores are too high. Conversely, for both the ‘moderate interest’ and ‘low interest’ scenarios, our scores are too low. Understanding overall societal values for species loss and determining the existence value of species diversity distinct from the ecological value of species diversity or the values from other services that species provide are both important areas for future research.

Habitat scores were generally lower in tropical developing countries, but were more variable among countries than species scores (Table S6; Fig. 3B). In fact, habitat scores were only very weakly correlated to species scores (R-squared  = 0.07; p-value <.0001). Several developed countries scored relatively low, including the United States, Spain, South Korea and France (Table S6; Fig. 3B), likely because their coastal areas have been highly developed and continue to be developed at a rapid rate. The highest scores for habitats included many countries and territories in the Pacific that are relatively isolated and therefore have relatively low levels of anthropogenic impact. Countries where likely future states may be worse compared to their current state included Somalia, Iran, Fiji, Angola, and Haiti (Table S6).

The relatively large number of countries with high habitat scores may have resulted from several factors including the habitats we assessed, the time period over which we measured change, and a lack of data from which to calculate status. Due to poor data availability, we were only able to assess habitat extent or condition for six habitats. Other habitats may have lower or higher status. The use of a 1980 s reference point could have resulted in higher scores for many developed countries where the coastal zone was modified before the reference point. Many developing countries have more recently been building in their coastal zones and may have been more likely to have declines detected than developed countries. In addition, data were sparse for many of the habitats that we did evaluate. For example, we had data for only 23% of the countries that are estimated to have salt marshes and we could not gap-fill because of the coarseness of the data. Therefore some countries may have salt marshes and may have experienced significant habitat losses, but we had no information on their extent and condition (or even their existence). Because we lacked geographically comprehensive data, countries may also have received higher scores where we did not have enough data to document habitat decline. For example, countries like Romania and Georgia likely had high habitat scores because we had limited data on their salt marshes and other coastal habitats. Forty-six percent of temperate countries (defined as countries with EEZs between −30° to −60°, +30° to +60°) had data only for subtidal soft-bottom from which to calculate habitat status. Subtidal soft-bottom was the only habitat where the pressures (i.e. intensity of trawl fishing) on the habitat were used as a proxy for its condition. In general, subtidal soft-bottom scores were relatively high and had little variability. Scores may be lower for temperate countries when better data on salt marshes and other temperate coastal habitats become available.

The status of individual habitats from our analyses indicate that salt marshes and sea ice had the worst current status and soft-bottom habitats had the best (Table 1). The scores of most of the individual habitats were positively correlated to the overall habitat scores (Table S8). The habitats that had the strongest correlations with the overall habitat score were mangroves, corals and seagrasses, followed by salt marshes and sea ice (Table S8). For most habitats, scores can be interpreted as a change in habitat extent, but for subtidal soft-bottom, differences related to the amount of pressure, making the connection to actual habitat degradation less direct. In addition, impacts on soft-bottom from illegal and unreported trawling could not be accounted for, so our soft-bottom scores may be overly optimistic. Salt marshes were underrepresented in the dataset, but where present had a large effect on goal scores, possibly due to the categorical values assigned to status and trend (i.e. −0.5, 0, and 1) because we lacked more quantitative data on condition. Mangroves and seagrasses had the strongest effects in tropical areas, followed by corals, which had a significant but weaker correlation. Values in temperate and subtropical regions were driven by seagrasses and salt marshes, although each of these habitats was present in less than half of the countries (Table S8). Boreal scores were driven by sea-ice, the most extensive habitat at high latitudes beside subtidal soft-bottom.

**Table 1:** Mean and standard deviation of status and trend scores for each habitat.

	Status (mean)	Status (sd)	Trend (mean)	Trend (sd)
corals	0.85	0.22	0.01	0.17
seagrasses	0.79	0.27	−0.35	0.95
mangroves	0.79	0.19	−0.59	0.49
salt marshes	0.69	0.25	0.19	0.25
soft-bottom	0.95	0.10	0.00	0.03
sea ice	0.71	0.33	−0.02	0.06

Although many marine habitats have undergone recent declines [30], [40], positive trends for some countries, including Canada, Russia, Australia, and many countries in Europe suggest that they have improved the condition of marine habitats within their EEZs since the reference time (∼ early 1980 s) that we used to assess current status. Trends remained generally negative across developing countries in the tropics, but were also negative in some developed countries, including the United States. Coral reef trends were nearly flat [28], [29], while seagrasses and mangroves had the largest declines (Table 1). Although salt marshes had the worst status, they had the most positive trend, which is possibly an artifact of using categorical values.

Quantifying how well biodiversity is currently doing depends on establishing the targets one hopes to achieve, so setting appropriate reference points for species and habitats was a critical component of calculating biodiversity scores [19]. There has been much attention to the concept of ‘baselines’ and ‘shifting baselines’ within marine ecosystems [47]–[49]. We know that many marine ecosystems have been fundamentally changed as marine populations have declined and exploitation pressures have increased over the last hundred years, particularly in recent decades [41], [47]. Regaining pristine conditions is not achievable in the foreseeable future, so we did not set reference points to historical or pristine abundance levels. Instead we set reference points that followed SMART principles – Specific, Measureable, Ambitious, Realistic and Time-bound [19], [50].

We established reference points for species based on the level of extinction risk from IUCN Red List species assessments [14], [16]. Our selected target was having all species at a status of Least Concern as defined by the IUCN Red List Categories and Criteria [14]. Importantly, a status of Least Concern does not require populations to be at pristine abundance levels, but instead to have a low risk of extinction globally. Because species assessments are conducted globally, population-level extinctions or increases would not necessarily be detected in this global assessment. However, national-scale Red List assessments could be included in regional-scale assessments of biodiversity using the method presented here, as was done for a regional application of the Ocean Health Index to Brazil. National-scale Red Lists and IUCN global assessments can have a relatively high level of agreement, at least in terrestrial ecosystems [51], but the wide distribution of many marine species and their complex population structure may result in more discrepancies.

No well-established target exists for reducing habitat loss. Common practice dictates setting a target at some time when the habitat was considered ‘intact’ or in relatively good condition. However, determining what is realistic and achievable in terms of habitat restoration remains a fundamental challenge. At least 30% of the original extent of seagrasses and mangroves have already been lost [30], [39] and coral reefs have lost nearly 80% of their cover in the Caribbean since the late 1980 s. Undoubtedly, these ecosystems had already experienced significant losses prior to the 1980 s [40], [52]. Particularly in coastal areas with high human population density, restoring habitats to ‘pristine’ levels is probably not possible, at least in the foreseeable future. We chose a general reference point of habitat extent in the 1980 s for most marine habitats. Satellite monitoring and SCUBA diving only began in the late 1970 s, so systematic global estimates for some datasets like mangroves and coral reefs do not have much sampling before the 1980 s. Therefore, habitat extent in the 1980 s represents a target that fulfills SMART principles, although there may be changes in habitat extent and condition that we were unable to capture.

**Figure 8:**
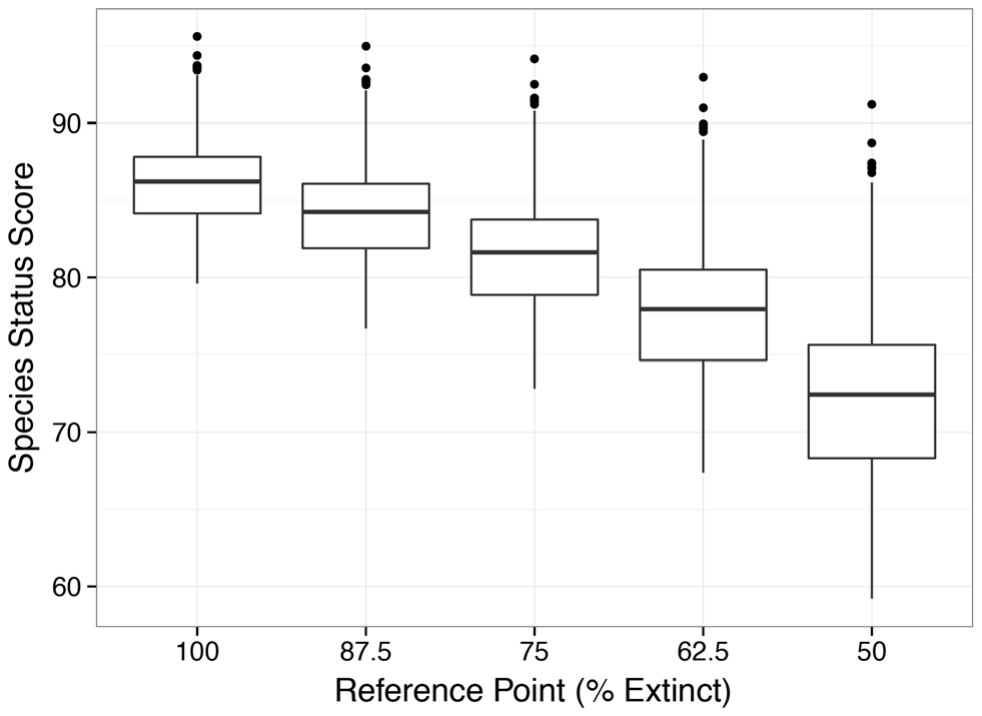
Distribution of species status scores by EEZ region when the lowest possible score was defined by different values of % of extinct species. The median value divides the box, which extends from the first (25%) to the third (75%) quartiles of the distribution. Whiskers extend to the last point within this interquartile range * 1.5 and dots indicate outliers.

In addition to the challenges of setting appropriate reference points, measuring the status of biodiversity is fundamentally affected by the form of the relationship between habitat or species loss and public perception of the impact of different levels of loss [45] and how extinction risk is calculated across species. For example, we measured relative risk instead of the number of species at risk. We also weighted all species equally rather than by taxonomic group. In addition, in the absence of better information, we assumed a linear relationship where the loss of each species is equally important. Species scores were also affected by how we scaled the lower boundary of status scores. [26]. With a lower-bound reference point of 50% instead of 75%, scores on average declined by 9 points. There is also evidence of a nonlinear decrease in the mean score as this lower-bound reference point changes from 100% to 50% (Fig. 8), driven by the number of species in the Near Threatened and Vulnerable categories (Fig. S8).

Although marine species and habitats support a vast amount of biodiversity, they are also highly dynamic and difficult to monitor at the global scale with the present level of surveying and the limits of current remote sensing techniques for marine ecosystems. We were limited taxonomically to the species that have been assessed for their extinction risk (Table S3; Fig. S1–S3). Most taxa in our analysis were tropical species. In temperate and boreal regions, pelagic species were principally represented by marine mammals, tunas and billfish, and sharks and rays. A few taxa (e.g. sharks and mammals) had the greatest effect on the status score when removed (Table S7). Nonetheless, our analyses suggest that the exclusion of different taxonomic groups changed scores by less than 1% on average, suggesting that no single species group is strongly driving our results (Table S7; Figs. S5, S6). Including more species as they are assessed and having more data on population trends will help to increase the accuracy of the species scores in the future.

We would have liked to have been able to assess other key habitats, including but not limited to oyster beds, rocky reefs, kelp forests, seamounts and pelagic habitats that support biodiversity, but global data were not available for them. Furthermore, we had to employ gap-filling measures or proxies for many of the marine habitat datasets that we did use. Datasets on salt marshes and seagrasses were some of the poorest in terms of coverage and quality, but are critical not only to biodiversity accounting, but also to accounting for carbon sequestration [53] and protection of coastlines from storms and erosion. Because the best habitat data currently available are for mangroves and tropical corals, it was easier to estimate condition for the tropics than for the temperate and boreal areas.

Because we rely on globally consistent data for our calculations, we could not assess the effectiveness of specific national or sub-national laws or the degree of enforcement in existing protected areas. We assumed that participation in international conventions related to biodiversity as well as overall governance metrics would be indicative of having the necessary legal and social structures to implement regulations related to resource management. This assumption is probably overly optimistic for some countries. Although our resilience measures can help to broadly convey whether governance to protect biodiversity is in place and highlight where effective management may be working, determining what may be the most effective measures for protecting species and habitats must be made at scales that are appropriate for management and account for local context.

Biodiversity is increasingly recognized for its existence value, its foundational importance to ecosystem structure and provision of ecosystem benefits and services that support human well-being. Our results provide the first country-by-country estimates for how species and habitats are faring and highlight key areas for data improvement so that biodiversity might be assessed more accurately in the future. Biodiversity is threatened in many countries, particularly along the west coast of Africa and in Southeast Asia. Lower resilience scores and higher pressure scores helped drive likely future state scores lower for many countries (Figs. 3E–H, 5). The strong relationship between resilience and HDI suggests that many less developed countries do not currently have the institutions in place to improve their biodiversity scores in the short-term (Fig. 6). Governance mechanisms will likely need to be strengthened not only in developing countries, but also in places currently scoring relatively well because species and habitats have negative trends in nearly all countries. Effective governance is widely recognized as essential for biodiversity conservation and resource management [54], [55], but management measures often must be in place for several years before positive effects are realized [56]–[58]. Therefore, establishing effective governance now should be a priority if countries are to mitigate further declines. Our results emphasize the importance of working towards filling key knowledge gaps and developing the institutions and regulations needed to meet CBD goals and sustain marine biodiversity for the long-term benefit of all life on earth.

## Supporting Information

Figure S1 **Number of species by taxonomic group for assessed, mapped and all catalogued species.** Catalogued numbers include many more species beyond the taxonomic group assessed. For example, only reef-building scleractinian corals, octocorals and hydrocorals were assessed, but Cnidaria include many other species including jellyfish, hydroids, and anemones. Of all species catalogued at the coarse taxonomic level, a subset of species has mapped distributions available from IUCN or Aquamaps [28], and a further subset of species has been assessed for extinction risk. Data corresponds to Table S5. Note that counts are given on a log-10 scale. The catalogued numbers are representative of the coarser taxonomic class listed on the far left.
(TIF)

Figure S2 **Species richness of assessed species within EEZs.** Mean species counts are provided across bands of latitude (1 to 342) and longitude (12 to 458) as a greyed histogram in the margins.
(TIF)

Figure S3 **Percent of species assessed within EEZs relative to those that have been mapped.** This map shows the number of species that have been assessed by IUCN out of all the marine species that have a distribution map from Aquamaps [28] or IUCN data [29]–[34]. Although nearly all species in the Arctic appear to have been assessed, these high numbers reflect only that most of the species that have been mapped have been assessed. Many species do not yet have distribution maps. The average percentages are shown in the grey histogram margins, which differ in range longitudinally (13 to 45%) and latitudinally (10 to 100%).
(TIF)

Figure S4 **Average extinction risk.** In our analysis we subtracted the weighted average of extinction risk from 1, and multiplied by 100. An average risk of 100 would mean all species are at Least Concern and a score of 0 would indicate all are Extinct. We did not include extinct species in our analysis, so the lowest possible score is 20 for all being Critically Endangered. The average percentages are shown in the grey histogram margins, which differ in range longitudinally (79 to 95) and latitudinally (77 to 100).
(TIF)

Figure S5 **Percent change in average extinction risk by excluding different taxa in a jacknife analysis.** A higher percent change means that excluding a particular taxon increased the recalculated average extinction risk by that much percentage of the original score (Figure S4). The range of differences was dominated by the exclusion of marine mammals, positively in the Arctic and negatively in the Antarctic. Inclusion of marine mammals therefore reduced the score in the Arctic and increases it in the Antarctic. Other pelagic taxa also appeared with subtler differences, and all coastal species except corals had too little differentiation to be visible.
(TIF)

Figure S6 **Mean percent difference between the status scores calculated across all taxa and the status scores obtained excluding one of the taxonomic groups.** Status scores were calculated for each region excluding each taxonomic group (Table S7) and the mean value for all these scores was taken. Then the difference between these values and the scores that included all taxa was calculated (Diff column of Table S7). In order to express them as percentages of the original calculated value, they were divided by the all-taxa status scores and multiplied by 100. The mean percent difference is a proxy for how much a given taxon affects the status score.
(TIF)

Figure S7 **Species status scores for EEZ regions by the four weighting schemes applied.** Weighting schemes are shown in Fig. 1. When Vulnerable and Endangered were weighted more heavily, as in Logistic B, the scores were lower.
(TIF)

Figure S8 **Histograms of IUCN extinction risk categories by number of species in each category.**
(TIF)

Table S1 **Pressures and weights used for species and habitats scores.** Each column is a pressure that was used in the model. For each habitat and for all species, the relative contribution of each of the ecological pressures to the overall pressure score was based on whether they were ranked as having ‘high’ (score  = 3), ‘medium’ (score  = 2) or ‘low’ (score  = 1) impact. Social pressures were based on a single index so no relative weights were applied. An ‘x’ denotes where they were factored in the calculation. The overall weighted ecological pressures contribute 50% of the overall pressure score and the overall social pressures contribute the other 50%. Detailed descriptions on the datasets used for pressures can be found in Halpern *et al.* [1].
(DOCX)

Table S2 **Resilience measures used for the species and habitats assessments.** Indicators that were used for each of the habitats or the species sub-goal are denoted with an ‘X’. Abbreviations in the table are as follows: The Convention on Biological Diversity (CBD), Convention on International Trade in Endangered Species of Wild Fauna and Flora (CITES), Worldwide Governance Indicators (WGI), and Exclusive Economic Zone (EEZ). Versions within the Fishing Resilience (EEZ) category refer to whether commercial fisheries management, artisanal fisheries management, or both types of fisheries management most influence the goal. Version 1 includes a measure of commercial only, Version 3 includes artisanal only, and Version 2 includes both commercial and artisanal fisheries management. Details on data sources and development are in Halpern *et al.* [1].
(DOCX)

Table S3 **Taxonomic groupings and counts for species in the analysis.** Species counts by extinction risk category were limited to those assessed with a defined geographic distribution available from either IUCN or the Aquamaps species distribution database [28]. Total unassessed numbers are derived from species in the Aquamaps species distribution database [28]. Extinction risk categories are as follows: Critically Endangered (CR), Endangered (EN), Vulnerable (VU), Near Threatened (NT), and Least Concern (LC). Total numbers differ slightly from Halpern *et al*. [1] because we did not include species that did not have a designated extinction risk assigned (i.e. 92 species had population trend and distribution, but not extinction risk).
(DOCX)

Table S4 **Weights used for weighted-average assessment of species, based on IUCN risk categories established by Butchart** ***et al*** **. 2007.**
(DOCX)

Table S5 **Number of catalogued, mapped and assessed species.** The assessed and mapped counts (see Table S3 for more detailed breakdown) are given by coarse taxon, and as percentage of species catalogued. All numbers come from Bouchet [24], except for Mammalia [25] and Reptilia [26].
(DOCX)

Table S6 **Score results for biodiversity (BD) and each dimension for the habitat (HAB) and species (SPP) calculations.**
(DOCX)

Table S7 **Species status per region and globally, with status scores recalculated excluding each taxon (Jackknife analysis).** The differences between the ‘all taxa included’ status score and the scores with each taxon excluded individually are also presented averaged across all countries (mean ±SD) and as a percent difference (i.e. divided by the all taxa status score × 100). Each column has the scores with a particular taxon excluded as follows: corals (Cor); hagfishes (Hag); mangroves (Man); marine mammals (Mar); other classes (Oth); reptiles (Rep); seagrasses (Sea); sharks, rays and skates (Sha); angelfish (fAn); butterflyfish (fBu); groupers (fGr); other fish (fOt); parrotfish (fPa); tunas & billfishes (fTu); wrasses (fWr). In order to compare taxonomic group effects by geographical area, the mean absolute difference (Diff) across excluded groups was obtained for each reporting region.
(DOCX)

Table S8 **Pearson adjusted correlation coefficients (adjusted r^2^) of the linear regression of overall habitat score versus individual habitat scores.** The correlations were obtained separately for reporting regions within three broad latitudinal ranges: tropical (TR, −30° to +30°), temperate and sub-tropical (TT, −30° to −60°, +30° to +60°) and boreal (BO, >60°, <−60°). The number of reporting regions is shown in parentheses. Correlations were excluded when the habitat occurred in less than 5 regions within that latitudinal range. Significance codes (p-values): <0.001 =  ‘***’, <0.01 =  ‘**’, <0.05 ‘*’, <0.1 = ‘.’, >0.1 = ‘ ’. See Table S6 for designation of latitudinal range.
(DOCX)

Text S1 **Supporting Methods and Results.**
(DOC)
