# Supplementary figures and images for: Assessing Global Marine Biodiversity Status within a Coupled Socio-Ecological Perspective
Source: PLoS One. 2013 Apr 11;8(4):e60284. doi: 10.1371/journal.pone.0060284 (PMC3623975; doi:10.1371/journal.pone.0060284)

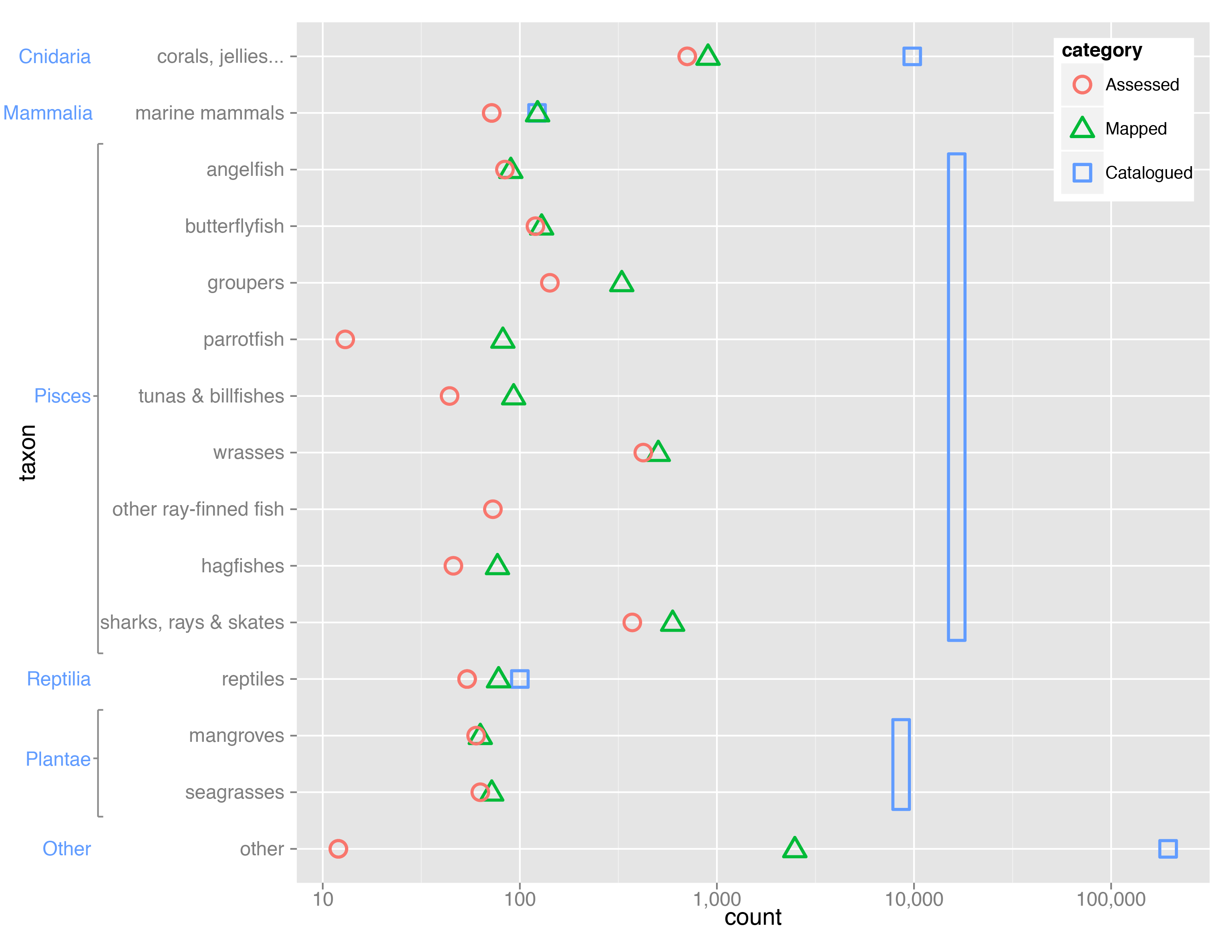

Supplement: Figure S1 — Number of species by taxonomic group for assessed, mapped and all catalogued species. Catalogued numbers include many more species beyond the taxonomic group assessed. For example, only reef-building scleractinian corals, octocorals and hydrocorals were assessed, but Cnidaria include many other species including jellyfish, hydroids, and anemones. Of all species catalogued at the coarse taxonomic level, a subset of species has mapped distributions available from IUCN or Aquamaps [28], and a further subset of species has been assessed for extinction risk. Data corresponds to Table S5. Note that counts are given on a log-10 scale. The catalogued numbers are representative of the coarser taxonomic class listed on the far left. (TIF) [file pone.0060284.s001.tif]

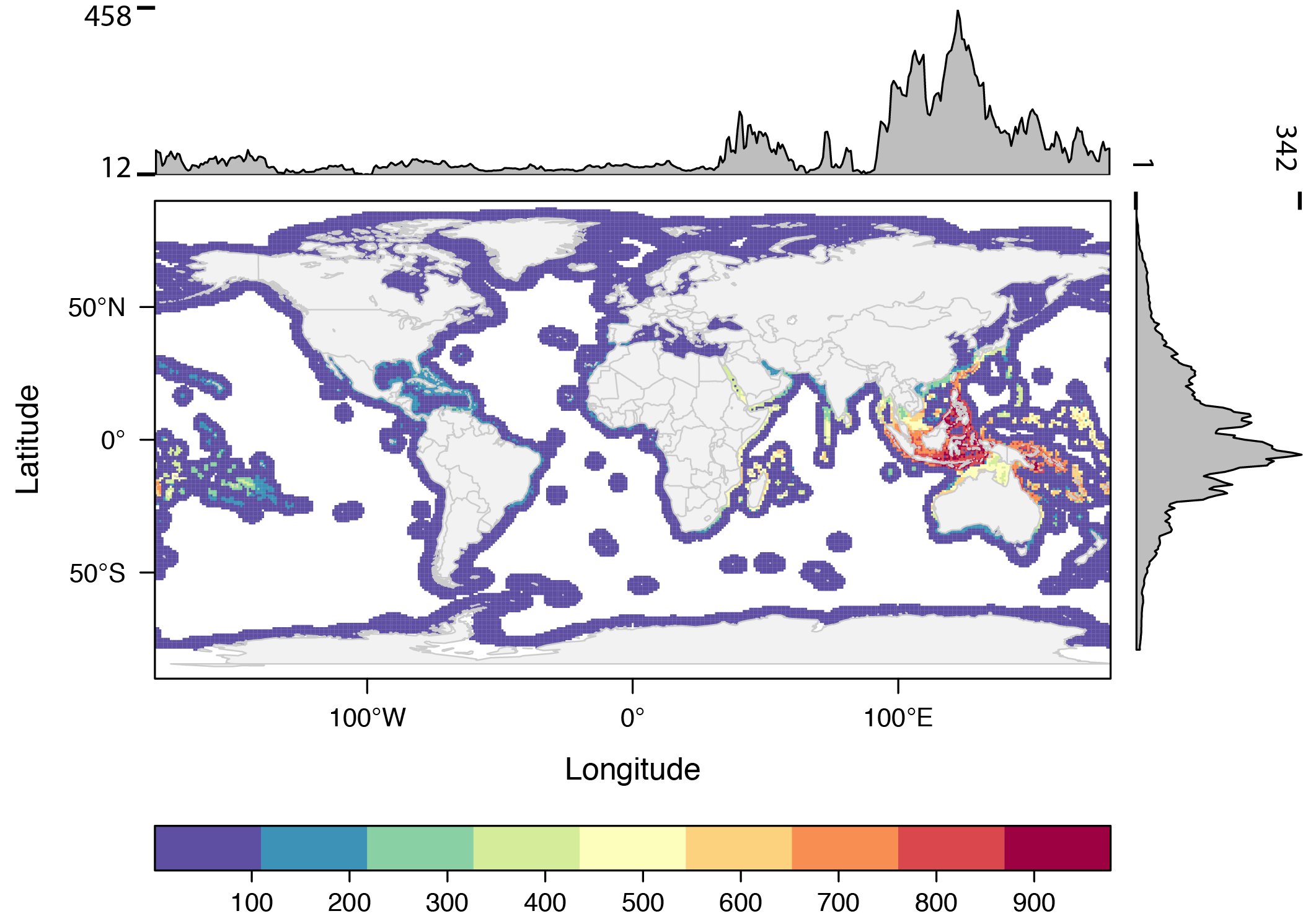

Supplement: Figure S2 — Species richness of assessed species within EEZs. Mean species counts are provided across bands of latitude (1 to 342) and longitude (12 to 458) as a greyed histogram in the margins. (TIF) [file pone.0060284.s002.tif]

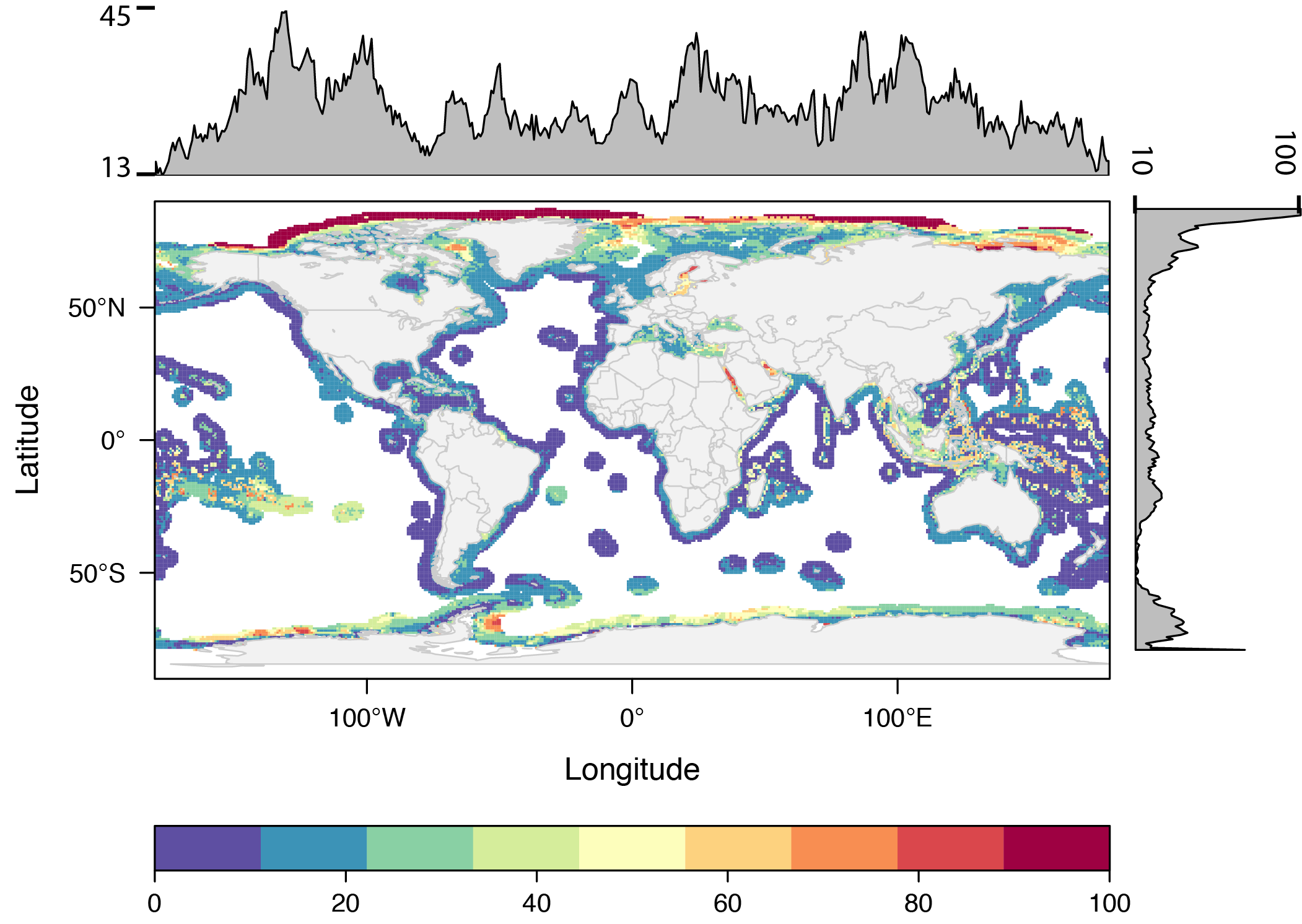

Supplement: Figure S3 — Percent of species assessed within EEZs relative to those that have been mapped. This map shows the number of species that have been assessed by IUCN out of all the marine species that have a distribution map from Aquamaps [28] or IUCN data [29]–[34]. Although nearly all species in the Arctic appear to have been assessed, these high numbers reflect only that most of the species that have been mapped have been assessed. Many species do not yet have distribution maps. The average percentages are shown in the grey histogram margins, which differ in range longitudinally (13 to 45%) and latitudinally (10 to 100%). (TIF) [file pone.0060284.s003.tif]

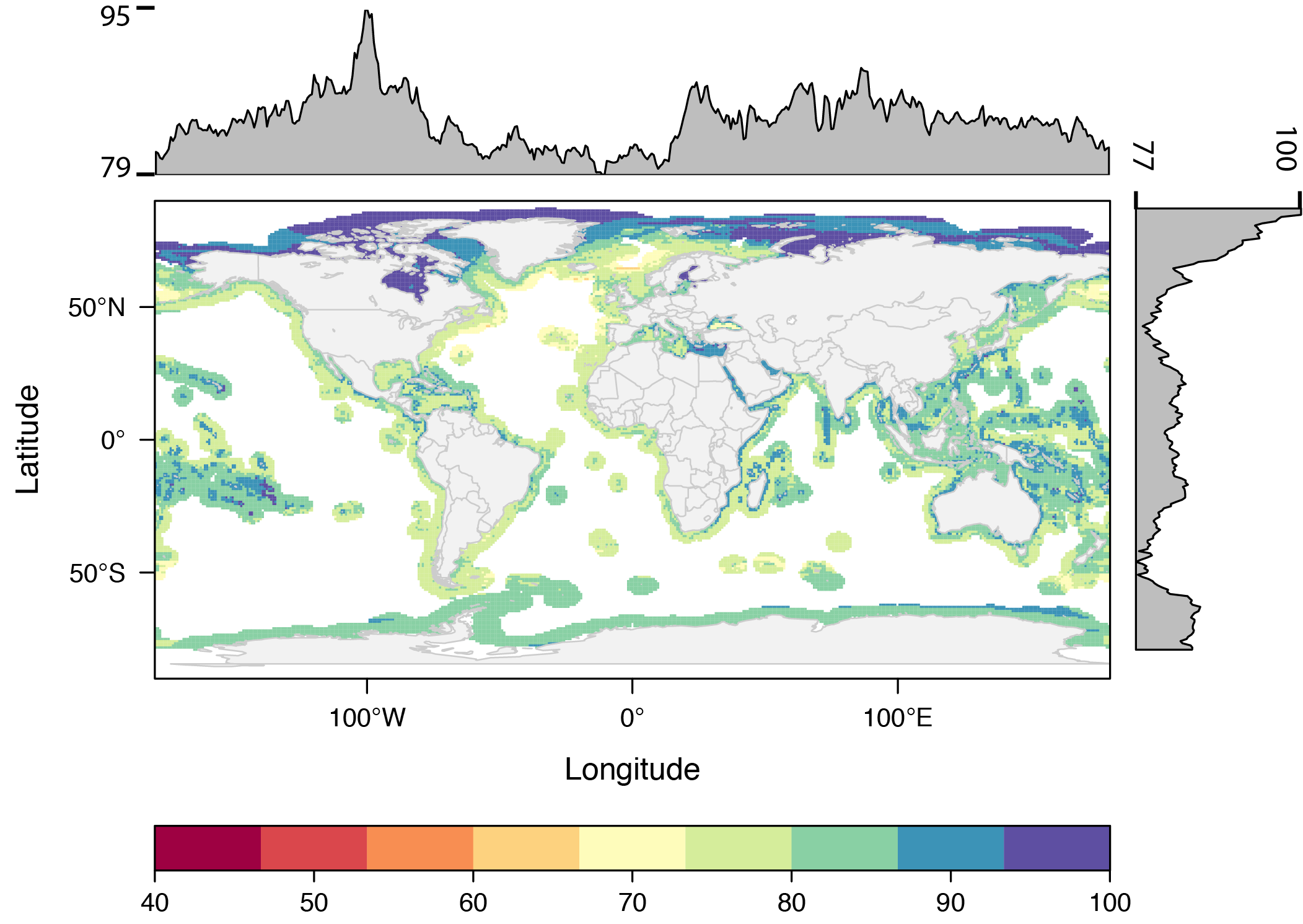

Supplement: Figure S4 — Average extinction risk. In our analysis we subtracted the weighted average of extinction risk from 1, and multiplied by 100. An average risk of 100 would mean all species are at Least Concern and a score of 0 would indicate all are Extinct. We did not include extinct species in our analysis, so the lowest possible score is 20 for all being Critically Endangered. The average percentages are shown in the grey histogram margins, which differ in range longitudinally (79 to 95) and latitudinally (77 to 100). (TIF) [file pone.0060284.s004.tif]

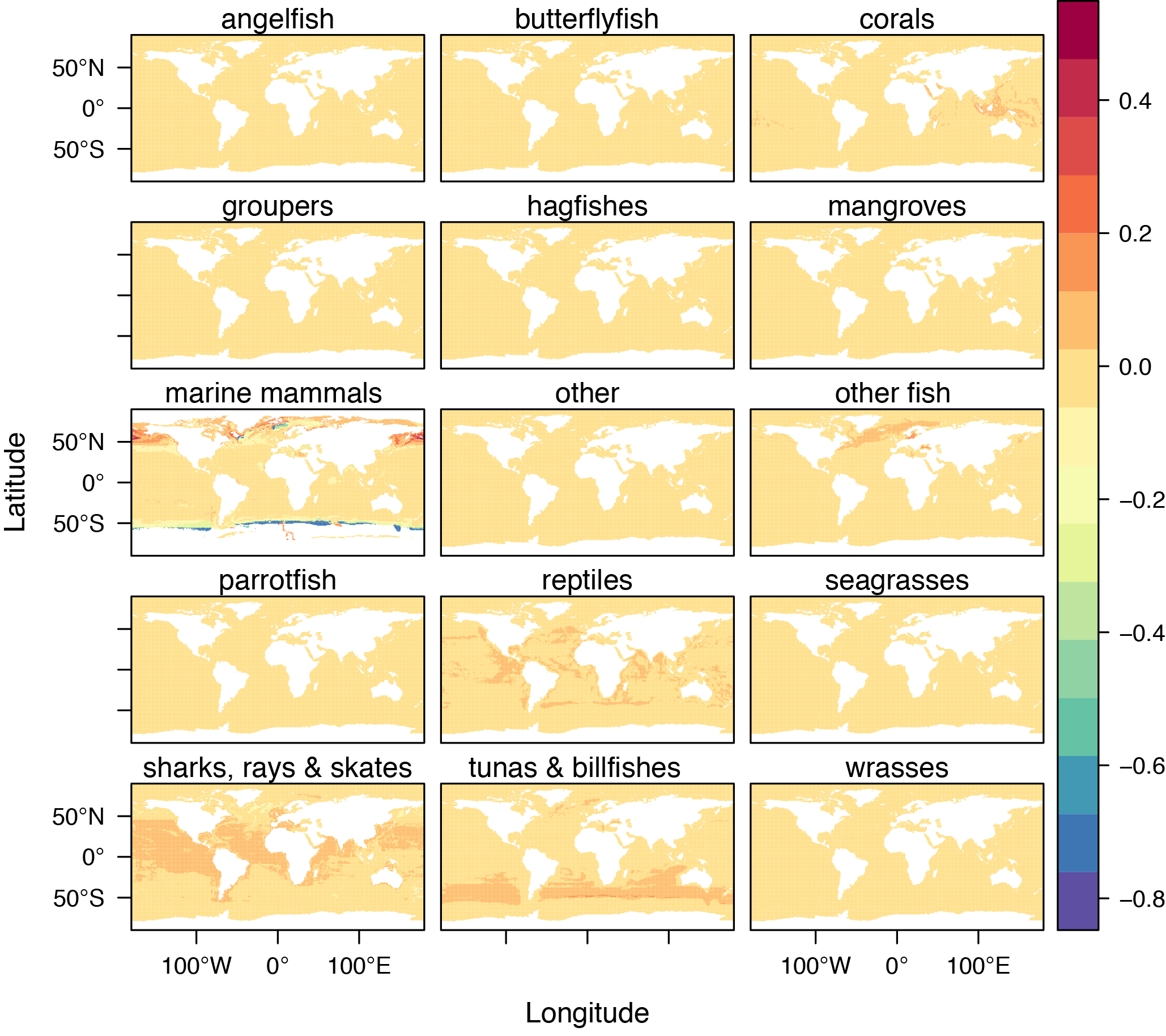

Supplement: Figure S5 — Percent change in average extinction risk by excluding different taxa in a jacknife analysis. A higher percent change means that excluding a particular taxon increased the recalculated average extinction risk by that much percentage of the original score (Figure S4). The range of differences was dominated by the exclusion of marine mammals, positively in the Arctic and negatively in the Antarctic. Inclusion of marine mammals therefore reduced the score in the Arctic and increases it in the Antarctic. Other pelagic taxa also appeared with subtler differences, and all coastal species except corals had too little differentiation to be visible. (TIF) [file pone.0060284.s005.tif]

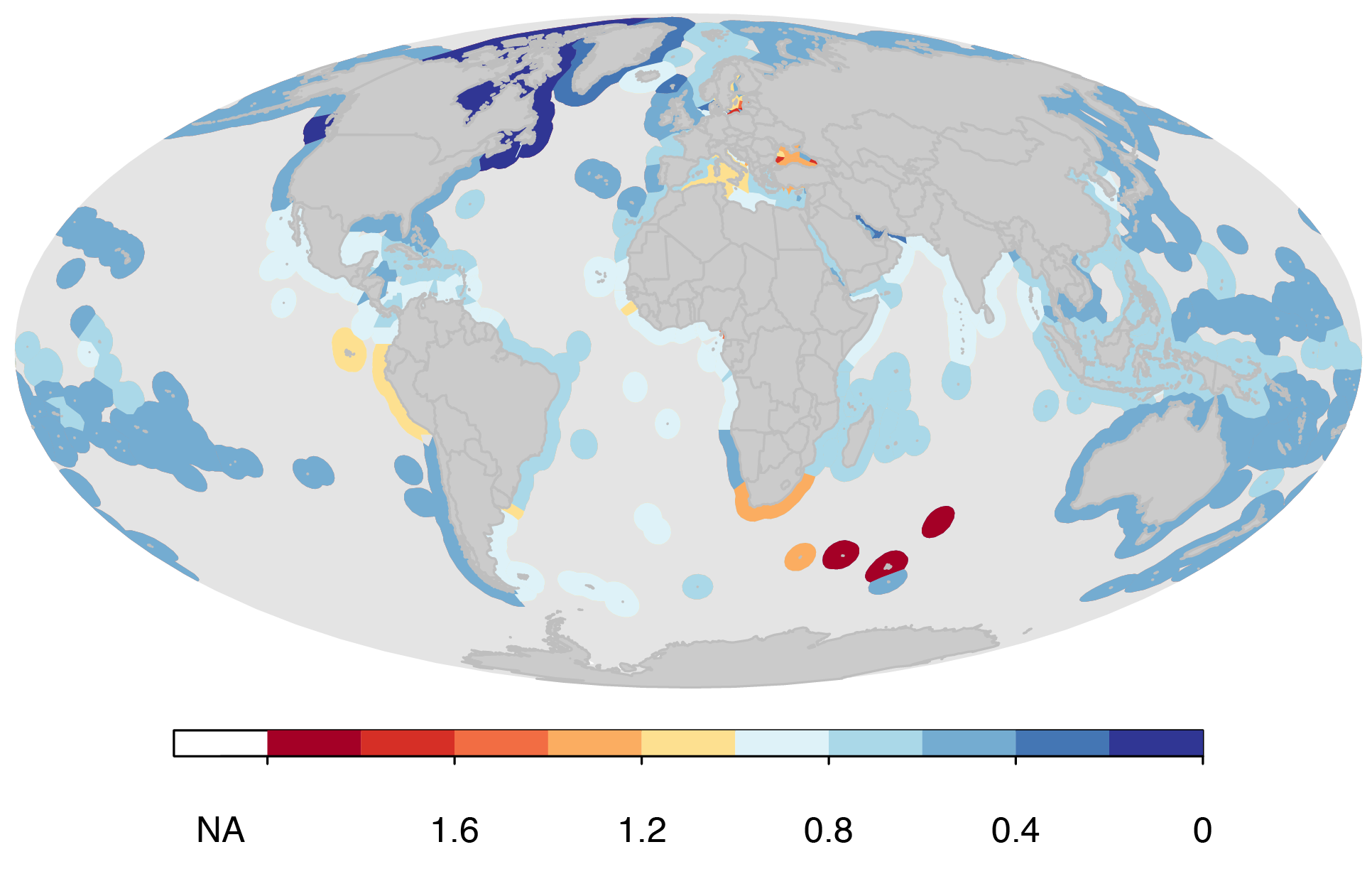

Supplement: Figure S6 — Mean percent difference between the status scores calculated across all taxa and the status scores obtained excluding one of the taxonomic groups. Status scores were calculated for each region excluding each taxonomic group (Table S7) and the mean value for all these scores was taken. Then the difference between these values and the scores that included all taxa was calculated (Diff column of Table S7). In order to express them as percentages of the original calculated value, they were divided by the all-taxa status scores and multiplied by 100. The mean percent difference is a proxy for how much a given taxon affects the status score. (TIF) [file pone.0060284.s006.tif]

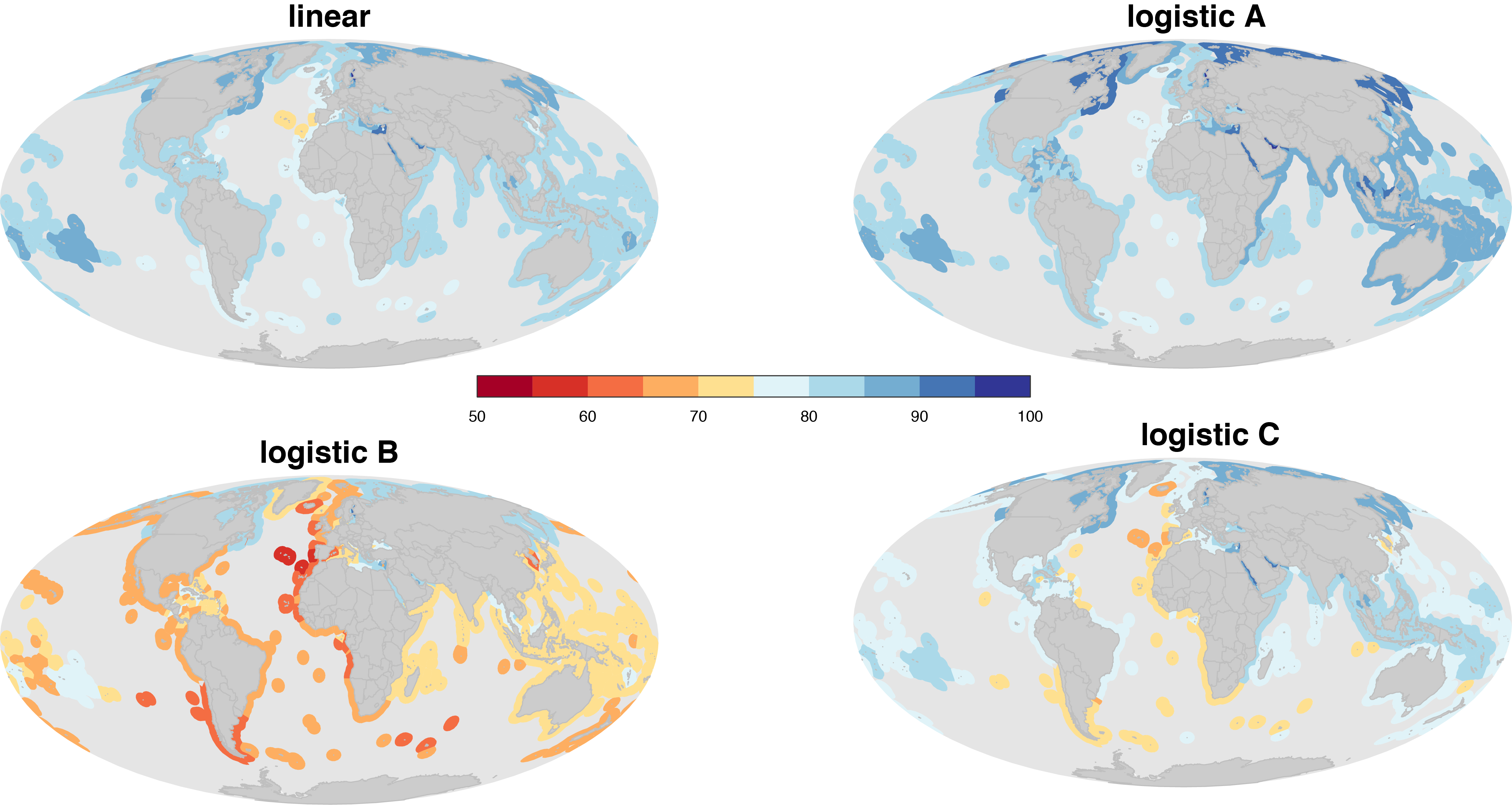

Supplement: Figure S7 — Species status scores for EEZ regions by the four weighting schemes applied. Weighting schemes are shown in Fig. 1. When Vulnerable and Endangered were weighted more heavily, as in Logistic B, the scores were lower. (TIF) [file pone.0060284.s007.tif]

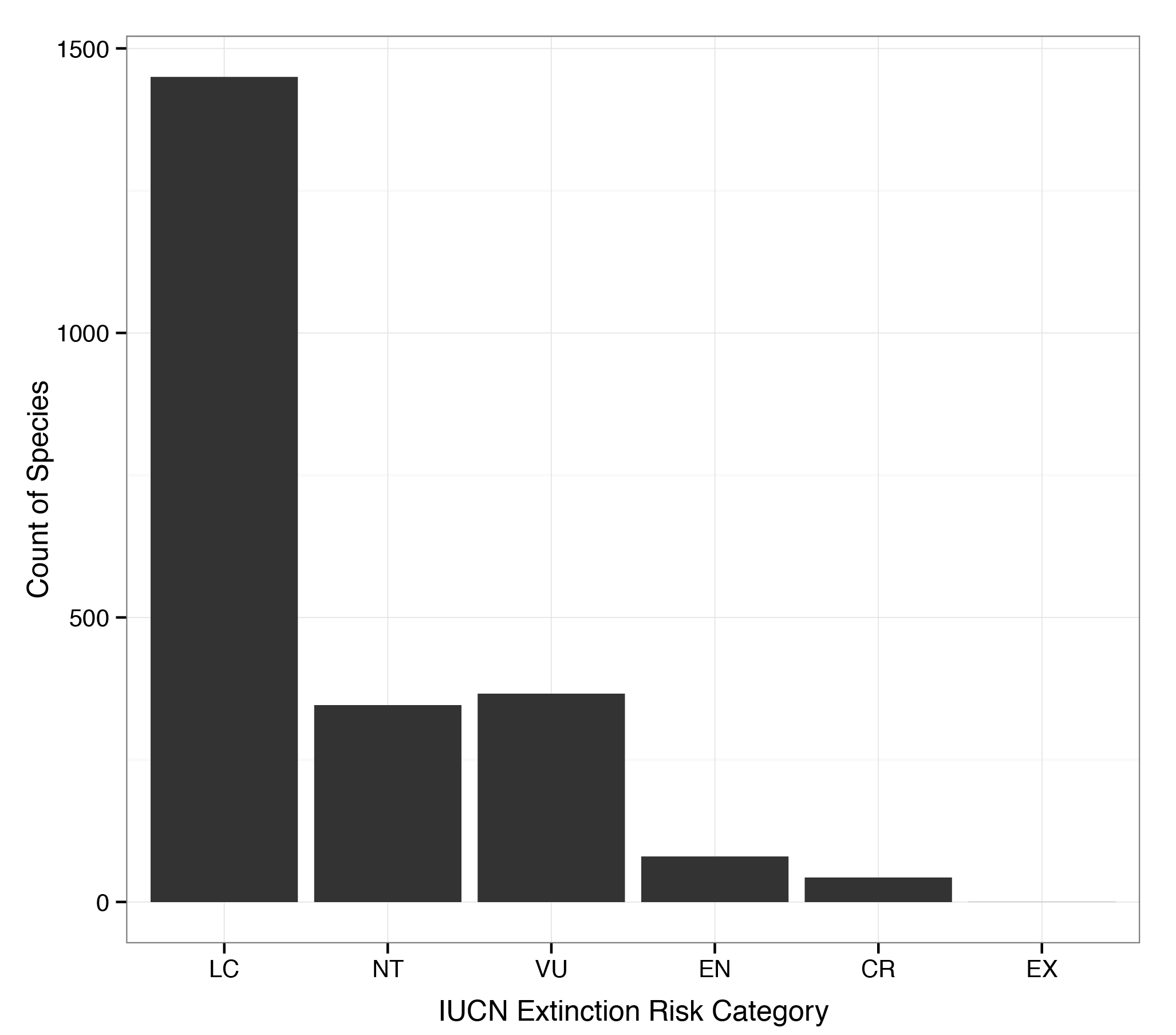

Supplement: Figure S8 — Histograms of IUCN extinction risk categories by number of species in each category. (TIF) [file pone.0060284.s008.tif]
