# Supplementary material for: Assessing Global Marine Biodiversity Status within a Coupled Socio-Ecological Perspective
Source: PLoS One. 2013 Apr 11;8(4):e60284. doi: 10.1371/journal.pone.0060284 (PMC3623975; doi:10.1371/journal.pone.0060284)
Supplement: Table S1 — Pressures and weights used for species and habitats scores. Each column is a pressure that was used in the model. For each habitat and for all species, the relative contribution of each of the ecological pressures to the overall pressure score was based on whether they were ranked as having ‘high’ (score = 3), ‘medium’ (score = 2) or ‘low’ (score = 1) impact. Social pressures were based on a single index so no relative weights were applied. An ‘x’ denotes where they were factored in the calculation. The overall weighted ecological pressures contribute 50% of the overall pressure score and the overall social pressures contribute the other 50%. Detailed descriptions on the datasets used for pressures can be found in Halpern et al. [1]. (DOCX) [file pone.0060284.s009.docx]

|  | **ECOLOGICAL PRESSURES** | | | | | | | | | | | | | | | | **SOCIAL PRESSURES** |
| --- | --- | --- | --- | --- | --- | --- | --- | --- | --- | --- | --- | --- | --- | --- | --- | --- | --- |
|  | **Water pollution** | | | | **Habitat destruction** | | | **Species introductions** | | **Fishing pressure** | | | | **Climate change** | | | **Social** |
| **SUB-GOAL** | Chemicals (incl. Oil) | Human Pathogens | Eutrophication & hypoxia | Trash | Subtidal softbottom (trawling, benthic structures) | Subtidal hardbottom (destructive artisanal) | Intertidal (land-sea interface) | Alien species | Genetic Escapes | Commercial high bycatch (demersal and pelagic) | Commercial low bycatch (demersal and pelagic) | Artisanal low bycatch (incl. cyanide) | Artisanal high bycatch (blast fishing) | Sea surface temperature | pH | Ultraviolet radiation | 1-Worldwide Governance Indicators (All 6) |
| **Habitats** |  |  |  |  |  |  |  |  |  |  |  |  |  |  |  |  |  |
| **Mangroves** | **1** |  | **1** |  |  |  | **3** |  |  |  |  |  |  |  |  |  | x |
| **Seagrasses** | **2** |  | **3** |  |  |  | **3** | **1** |  |  |  |  |  | **2** | **1** |  | x |
| **Salt marshes** | **1** |  | **2** |  |  |  | **3** | **1** |  |  |  |  |  |  |  |  | x |
| **Sub-tidal soft bottom** | **2** |  | **2** |  | **3** |  |  | **1** |  | **3** | **1** | **1** |  |  |  |  | x |
| **Corals** | **1** |  | **2** |  |  | **3** |  | **1** |  |  |  |  | **3** | **3** | **1** | **1** | x |
| **Sea-ice** |  |  |  |  |  |  |  |  |  |  |  |  |  | **3** |  |  | x |
| **Species** | **2** |  | **3** | **1** | **3** | **2** | **2** | **1** | **1** | **3** | **1** | **1** | **2** | **1** | **1** | **1** | x |
