# Supplementary material for: Assessing Global Marine Biodiversity Status within a Coupled Socio-Ecological Perspective
Source: PLoS One. 2013 Apr 11;8(4):e60284. doi: 10.1371/journal.pone.0060284 (PMC3623975; doi:10.1371/journal.pone.0060284)
Supplement: Table S2 — Resilience measures used for the species and habitats assessments. Indicators that were used for each of the habitats or the species sub-goal are denoted with an ‘X’. Abbreviations in the table are as follows: The Convention on Biological Diversity (CBD), Convention on International Trade in Endangered Species of Wild Fauna and Flora (CITES), Worldwide Governance Indicators (WGI), and Exclusive Economic Zone (EEZ). Versions within the Fishing Resilience (EEZ) category refer to whether commercial fisheries management, artisanal fisheries management, or both types of fisheries management most influence the goal. Version 1 includes a measure of commercial only, Version 3 includes artisanal only, and Version 2 includes both commercial and artisanal fisheries management. Details on data sources and development are in Halpern et al. [1]. (DOCX) [file pone.0060284.s010.docx]

|  |  |  |  |  |  |  |  |  |  |
| --- | --- | --- | --- | --- | --- | --- | --- | --- | --- |
|  |  |  |  |  |  |  |  |  |  |
| **SUB-GOAL** | **CBD Water** | **Habitat Resilience (EEZ)** | **Fishing Resilience (EEZ)** | **CBD Tourism** | **CBD Mariculture** | **CBD Alien Sp.** | **CITES Signatories** | **Diversity Index (EEZ)** | **WGI (all 6 indicators)** |
| **Habitat** |  |  |  |  |  |  |  |  |  |
| Mangroves |  | **X** |  | **X** | **X** | **X** |  | **X** | **X** |
| Seagrasses | **X** | **X** |  | **X** | **X** | **X** |  | **X** | **X** |
| Salt marshes | **X** | **X** |  | **X** | **X** | **X** |  | **X** | **X** |
| Sub-tidal soft bottom | **X** | **X** | **VERSION 1** | **X** | **X** | **X** |  | **X** | **X** |
| Corals | **X** | **X** | **VERSION 3** | **X** | **X** | **X** |  | **X** | **X** |
| Sea Ice |  |  |  | **X** | **X** | **X** |  | **X** | **X** |
| **Species** | **X** | **X** | **VERSION 2** | **X** | **X** | **X** | **X** |  | **X** |
