# Supplementary material for: Assessing Global Marine Biodiversity Status within a Coupled Socio-Ecological Perspective
Source: PLoS One. 2013 Apr 11;8(4):e60284. doi: 10.1371/journal.pone.0060284 (PMC3623975; doi:10.1371/journal.pone.0060284)
Supplement: Table S3 — Taxonomic groupings and counts for species in the analysis. Species counts by extinction risk category were limited to those assessed with a defined geographic distribution available from either IUCN or the Aquamaps species distribution database [28]. Total unassessed numbers are derived from species in the Aquamaps species distribution database [28]. Extinction risk categories are as follows: Critically Endangered (CR), Endangered (EN), Vulnerable (VU), Near Threatened (NT), and Least Concern (LC). Total numbers differ slightly from Halpern et al. [1] because we did not include species that did not have a designated extinction risk assigned (i.e. 92 species had population trend and distribution, but not extinction risk). (DOCX) [file pone.0060284.s011.docx]

|  |  |  |  | **Extinction Risk** | | | | | **Total Assessed** | **Total Unassessed** | **% Assessed** |
| --- | --- | --- | --- | --- | --- | --- | --- | --- | --- | --- | --- |
| **Kingdom** | **Taxon** | **Common name** | **Class [> Order > Family]** | **CR** | **EN** | **VU** | **NT** | **LC** |  |  |  |
| Animalia | Cnidaria | corals | Anthozoa / Hydrozoa | 5 | 25 | 202 | 177 | 298 | 707 | 194 | 78.5 |
|  | Mammalia | marine mammals | Mammalia | 3 | 10 | 12 | 7 | 40 | 72 | 51 | 58.5 |
|  | Pisces | angelfish | Actinopterygii > Perciformes > Pomacanthidae | 0 | 1 | 1 | 2 | 80 | 84 | 6 | 93.3 |
|  |  | butterflyfish | Actinopterygii > Perciformes > Chaetodontidae | 0 | 0 | 0 | 3 | 117 | 120 | 9 | 93.0 |
|  |  | groupers | Actinopterygii > Perciformes > Serranidae | 4 | 5 | 14 | 23 | 96 | 142 | 187 | 43.2 |
|  |  | parrotfish | Actinopterygii > Perciformes > Odacidae / Scaridae | 0 | 0 | 2 | 0 | 11 | 13 | 69 | 15.9 |
|  |  | tunas & billfishes | Actinopterygii > Perciformes > Scombridae | 2 | 2 | 4 | 3 | 33 | 44 | 49 | 47.3 |
|  |  | wrasses | Actinopterygii > Perciformes > Labridae | 1 | 1 | 17 | 4 | 400 | 423 | 81 | 83.9 |
|  |  | other fish | Actinopterygii > other ray-finned fishes | 5 | 5 | 19 | 11 | 33 | 73 | 7,006 | 1.0 |
|  |  | hagfishes | Myxini | 1 | 2 | 6 | 2 | 35 | 46 | 31 | 59.7 |
|  |  | sharks, rays and skates | Chondrichthyes | 14 | 20 | 71 | 95 | 172 | 372 | 224 | 62.4 |
|  | Reptilia | reptiles | Reptilia | 5 | 3 | 4 | 4 | 38 | 54 | 24 | 69.2 |
| Plantae | Plants | seagrasses | Liliopsida | 0 | 3 | 7 | 5 | 48 | 63 | 9 | 87.5 |
|  |  | mangroves | Magnoliopsida / Polypodiopsida | 2 | 3 | 5 | 6 | 44 | 60 | 3 | 95.2 |
| Other | Other | other classes | Other Classes | 1 | 0 | 2 | 4 | 5 | 12 | 2,472 | 0.5 |
|  |  |  | **TOTAL** | 43 | 80 | 366 | 346 | 1,450 | 2,285 | 10,415 | 18.0 |
