# Supplementary material for: Assessing Global Marine Biodiversity Status within a Coupled Socio-Ecological Perspective
Source: PLoS One. 2013 Apr 11;8(4):e60284. doi: 10.1371/journal.pone.0060284 (PMC3623975; doi:10.1371/journal.pone.0060284)
Supplement: Table S4 — Weights used for weighted-average assessment of species, based on IUCN risk categories established by Butchart et al . 2007. (DOCX) [file pone.0060284.s012.docx]

| **Risk Category** | **IUCN code** | **Weight** |
| --- | --- | --- |
| Extinct | EX | 1.0 |
| Critically Endangered | CR | 0.8 |
| Endangered | EN | 0.6 |
| Vulnerable | VU | 0.4 |
| Near Threatened | NT | 0.2 |
| Least Concern | LC | 0.0 |
