# Supplementary material for: Assessing Global Marine Biodiversity Status within a Coupled Socio-Ecological Perspective
Source: PLoS One. 2013 Apr 11;8(4):e60284. doi: 10.1371/journal.pone.0060284 (PMC3623975; doi:10.1371/journal.pone.0060284)
Supplement: Table S5 — Number of catalogued, mapped and assessed species. The assessed and mapped counts (see Table S3 for more detailed breakdown) are given by coarse taxon, and as percentage of species catalogued. All numbers come from Bouchet [24], except for Mammalia [25] and Reptilia [26]. (DOCX) [file pone.0060284.s013.docx]

| **Taxon** | **Catalogued** | **Mapped (%)** | **Assessed (%)** |
| --- | --- | --- | --- |
| Cnidaria | 9,795 | 901 (9.2) | 707 (7.2) |
| Mammalia | 123 | 123 (100) | 72 (58.5) |
| Pisces | 16,475 | 8,979 (54.5) | 1,317 (8) |
| Reptilia | 100 | 78 (78) | 54 (54) |
| Plantae | 8,600 | 135 (1.6) | 123 (1.4) |
| Other | 194,622 | 2,484 (1.3) | 12 (0.01) |
| Total | 229,715 | 12,700 (5.5) | 2,285 (0.99) |
