# Supplementary material for: Assessing Global Marine Biodiversity Status within a Coupled Socio-Ecological Perspective
Source: PLoS One. 2013 Apr 11;8(4):e60284. doi: 10.1371/journal.pone.0060284 (PMC3623975; doi:10.1371/journal.pone.0060284)
Supplement: Table S6 — Score results for biodiversity (BD) and each dimension for the habitat (HAB) and species (SPP) calculations. (DOCX) [file pone.0060284.s014.docx]

| **EEZ region** | **Region** | **Status (HAB)** | **Trend (HAB)** | **Pressures (HAB)** | **Resilience (HAB)** | **Likely Future State (HAB)** | **Habitat Score (HAB)** | **Status (SPP)** | **Trend (SPP)** | **Pressures (SPP)** | **Resilience (SPP)** | **Likely Future State (SPP)** | **Species Score (SPP)** | **Biodiversity Score (BD)** |
| --- | --- | --- | --- | --- | --- | --- | --- | --- | --- | --- | --- | --- | --- | --- |
| Albania | TT | 100 | 0.02 | 32 | 59 | 100 | 100 | 83 | -0.2 | 42 | 54 | 76 | 80 | 90 |
| Algeria | TT | 88 | -0.02 | 44 | 51 | 90 | 89 | 80 | -0.21 | 51 | 47 | 68 | 74 | 82 |
| Angola | TR | 84 | -0.51 | 40 | 49 | 58 | 71 | 75 | -0.28 | 46 | 39 | 60 | 67 | 69 |
| Antigua and Barbuda | TR | 84 | -0.11 | 28 | 68 | 89 | 86 | 81 | -0.18 | 28 | 63 | 81 | 81 | 84 |
| Argentina | TT | 93 | 0 | 35 | 56 | 100 | 96 | 77 | -0.17 | 40 | 51 | 71 | 74 | 85 |
| Australia | TR | 89 | 0.29 | 15 | 80 | 100 | 94 | 82 | -0.34 | 17 | 78 | 79 | 80 | 87 |
| Australian Southern Ocean Territories | TR | 100 | 0 | 10 | 79 | 100 | 100 | 79 | -0.23 | 13 | 78 | 84 | 81 | 91 |
| Australian Tropical Territories | TR | 71 | -0.02 | 17 | 80 | 85 | 78 | 79 | -0.17 | 17 | 78 | 86 | 82 | 80 |
| Bahamas | TR | 79 | -0.34 | 21 | 72 | 74 | 77 | 83 | -0.16 | 25 | 67 | 85 | 84 | 80 |
| Bahrain | TR | 84 | -0.45 | 31 | 61 | 67 | 75 | 91 | -0.39 | 37 | 46 | 70 | 81 | 78 |
| Bangladesh | TR | 100 | 0.28 | 46 | 52 | 100 | 100 | 86 | -0.24 | 53 | 46 | 70 | 78 | 89 |
| Barbados | TR | 75 | 0.02 | 29 | 73 | 87 | 81 | 79 | -0.2 | 26 | 69 | 79 | 79 | 80 |
| Belgium | TT | 97 | 0.24 | 25 | 75 | 100 | 99 | 82 | -0.2 | 31 | 71 | 82 | 82 | 90 |
| Belize | TR | 54 | 0.18 | 34 | 61 | 66 | 60 | 84 | -0.16 | 38 | 57 | 80 | 82 | 71 |
| Benin | TR | 100 |  | 58 | 58 | 100 | 100 | 78 | -0.22 | 43 | 52 | 69 | 74 | 87 |
| Bosnia and Herzegovina | TT | 70 | -0.02 | 45 | 53 | 71 | 71 | 83 | -0.2 | 51 | 46 | 70 | 77 | 74 |
| Brazil | TR | 96 | -0.29 | 33 | 63 | 87 | 92 | 79 | -0.22 | 34 | 60 | 75 | 77 | 84 |
| British Caribbean Territories | TR | 87 | -0.3 | 22 | 71 | 84 | 86 | 78 | -0.19 | 24 | 68 | 79 | 78 | 82 |
| British Indian Ocean Territory | TR | 100 | 0 | 37 | 55 | 100 | 100 | 79 | -0.34 | 38 | 52 | 65 | 72 | 86 |
| British Pacific Territories (Pitcairn) | TR | 100 |  | 35 | 65 | 100 | 100 | 82 | -0.22 | 28 | 61 | 78 | 80 | 90 |
| British Southern Ocean Territories | TR | 89 | -0.02 | 13 | 76 | 100 | 95 | 77 | -0.13 | 17 | 74 | 85 | 81 | 88 |
| Bulgaria | TT | 100 | 0 | 28 | 58 | 100 | 100 | 77 | -0.23 | 34 | 51 | 70 | 73 | 87 |
| Cambodia | TR | 91 | -0.41 | 40 | 54 | 71 | 81 | 89 | -0.36 | 52 | 49 | 66 | 77 | 79 |
| Cameroon | TR | 89 | -0.23 | 41 | 52 | 79 | 84 | 78 | -0.22 | 51 | 48 | 66 | 72 | 78 |
| Canada | BO | 86 | 0 | 12 | 79 | 100 | 93 | 88 | -0.08 | 16 | 74 | 100 | 94 | 93 |
| Cape Verde | TR | 99 | -0.01 | 22 | 63 | 100 | 99 | 75 | -0.24 | 31 | 58 | 70 | 73 | 86 |
| Chile | TT | 84 | -0.09 | 19 | 76 | 95 | 90 | 77 | -0.18 | 26 | 75 | 81 | 79 | 84 |
| China | TR | 77 | -0.2 | 46 | 59 | 70 | 74 | 82 | -0.29 | 56 | 56 | 66 | 74 | 74 |
| Colombia | TR | 66 | -0.43 | 38 | 57 | 51 | 59 | 80 | -0.18 | 39 | 53 | 74 | 77 | 68 |
| Comoros | TR | 87 | 0 | 42 | 49 | 89 | 88 | 81 | -0.37 | 47 | 44 | 60 | 71 | 79 |
| Costa Rica | TR | 88 | -0.33 | 26 | 69 | 81 | 84 | 79 | -0.18 | 28 | 66 | 80 | 80 | 82 |
| Croatia | TT | 97 | 0.01 | 29 | 65 | 100 | 99 | 83 | -0.21 | 37 | 60 | 78 | 80 | 89 |
| Cuba | TR | 75 | 0.06 | 42 | 56 | 82 | 78 | 83 | -0.15 | 44 | 53 | 77 | 80 | 79 |
| Cyprus | TT | 93 | 0.03 | 20 | 76 | 100 | 97 | 90 | -0.14 | 27 | 72 | 95 | 93 | 95 |
| Democratic Republic of the Congo | TR | 65 | -0.5 | 54 | 42 | 41 | 53 | 85 | -0.21 | 64 | 35 | 65 | 75 | 64 |
| Denmark | BO | 91 | 0.02 | 19 | 76 | 100 | 96 | 83 | 0.01 | 21 | 73 | 98 | 90 | 93 |
| Djibouti | TR | 99 | 0 | 38 | 55 | 100 | 100 | 89 | -0.36 | 43 | 47 | 68 | 79 | 89 |
| Dominica | TR | 59 | -0.13 | 32 | 68 | 61 | 60 | 81 | -0.17 | 31 | 63 | 80 | 81 | 70 |
| Dominican Republic | TR | 87 | -0.32 | 40 | 59 | 74 | 80 | 82 | -0.16 | 41 | 55 | 77 | 79 | 80 |
| East Timor | TR | 82 | -0.4 | 39 | 49 | 63 | 72 | 86 | -0.39 | 45 | 35 | 60 | 73 | 73 |
| Ecuador | TR | 90 | -0.52 | 39 | 53 | 63 | 76 | 79 | -0.21 | 43 | 49 | 70 | 74 | 75 |
| Egypt | TR | 95 | 0 | 38 | 60 | 100 | 98 | 90 | -0.36 | 43 | 54 | 71 | 81 | 89 |
| El Salvador | TR | 80 | -0.5 | 35 | 61 | 60 | 70 | 81 | -0.18 | 39 | 59 | 76 | 78 | 74 |
| Equatorial Guinea | TR | 97 | -0.16 | 42 | 43 | 87 | 92 | 77 | -0.24 | 47 | 36 | 62 | 70 | 81 |
| Eritrea | TR | 95 | -0.11 | 45 | 48 | 88 | 92 | 89 | -0.41 | 49 | 40 | 62 | 76 | 84 |
| Estonia | TT | 96 | 0.15 | 20 | 73 | 100 | 98 | 89 | -0.14 | 28 | 67 | 92 | 90 | 94 |
| Fiji | TR | 91 | -0.48 | 40 | 49 | 64 | 78 | 83 | -0.35 | 41 | 41 | 63 | 73 | 75 |
| Finland | BO | 94 | -0.03 | 15 | 84 | 100 | 97 | 94 | -0.17 | 23 | 79 | 100 | 97 | 97 |
| France | TT | 62 | 0.24 | 26 | 75 | 82 | 72 | 78 | -0.24 | 30 | 74 | 77 | 77 | 75 |
| French Caribbean Territories | TR | 93 | -0.04 | 31 | 69 | 100 | 96 | 81 | -0.18 | 31 | 64 | 80 | 80 | 88 |
| French Guiana | TR | 100 | 0 | 17 | 70 | 100 | 100 | 78 | -0.23 | 23 | 67 | 77 | 78 | 89 |
| French Indian Ocean Territories | TR | 93 | 0.03 | 25 | 69 | 100 | 96 | 81 | -0.33 | 26 | 64 | 73 | 77 | 87 |
| French Polynesia | TR | 95 | 0 | 28 | 65 | 100 | 98 | 84 | -0.25 | 29 | 60 | 79 | 82 | 90 |
| French Southern Ocean Territories | TT | 100 | 0 | 11 | 75 | 100 | 100 | 74 | -0.01 | 17 | 74 | 87 | 80 | 90 |
| Gabon | TR | 65 | -0.52 | 36 | 49 | 45 | 55 | 76 | -0.25 | 45 | 43 | 63 | 69 | 62 |
| Gambia | TR | 85 | -0.45 | 41 | 57 | 64 | 75 | 76 | -0.24 | 47 | 55 | 66 | 71 | 73 |
| Georgia | TT | 100 | 0 | 31 | 55 | 100 | 100 | 80 | -0.23 | 39 | 48 | 70 | 75 | 88 |
| Germany | TT | 94 | 0.2 | 21 | 77 | 100 | 97 | 81 | -0.22 | 32 | 74 | 80 | 80 | 89 |
| Ghana | TR | 87 | -0.52 | 34 | 61 | 65 | 76 | 78 | -0.23 | 39 | 57 | 71 | 75 | 75 |
| Gibraltar | TT | 100 | 0 | 30 | 68 | 100 | 100 | 78 | -0.24 | 41 | 64 | 71 | 74 | 87 |
| Greece | TT | 92 | -0.02 | 26 | 69 | 100 | 96 | 86 | -0.18 | 34 | 65 | 85 | 85 | 91 |
| Grenada | TR | 64 | -0.5 | 30 | 65 | 50 | 57 | 83 | -0.17 | 34 | 59 | 81 | 82 | 69 |
| Guatemala | TR | 97 | -0.15 | 39 | 55 | 92 | 95 | 79 | -0.19 | 43 | 51 | 71 | 75 | 85 |
| Guinea | TR | 72 | -0.19 | 47 | 44 | 62 | 67 | 76 | -0.25 | 53 | 40 | 60 | 68 | 67 |
| Guinea-Bissau | TR | 67 | -0.5 | 41 | 49 | 47 | 57 | 76 | -0.25 | 47 | 45 | 63 | 69 | 63 |
| Guyana | TR | 93 | -0.29 | 33 | 55 | 81 | 87 | 78 | -0.24 | 39 | 50 | 69 | 73 | 80 |
| Haiti | TR | 79 | -0.51 | 46 | 47 | 52 | 66 | 83 | -0.15 | 48 | 33 | 71 | 77 | 71 |
| Honduras | TR | 85 | -0.31 | 42 | 57 | 72 | 79 | 83 | -0.17 | 42 | 54 | 76 | 79 | 79 |
| Iceland | BO | 63 | -0.06 | 19 | 70 | 71 | 67 | 74 | -0.13 | 25 | 64 | 77 | 75 | 71 |
| India | TR | 90 | -0.14 | 41 | 62 | 87 | 89 | 82 | -0.32 | 43 | 59 | 69 | 76 | 82 |
| Indonesia | TR | 84 | -0.5 | 36 | 59 | 63 | 74 | 84 | -0.39 | 43 | 55 | 65 | 74 | 74 |
| Iran | TR | 88 | -0.5 | 45 | 50 | 60 | 74 | 89 | -0.37 | 52 | 44 | 64 | 76 | 75 |
| Iraq | TR | 93 | 0 | 60 | 43 | 88 | 90 | 91 | -0.38 | 67 | 28 | 56 | 74 | 82 |
| Ireland | TT | 72 | 0.01 | 22 | 76 | 85 | 78 | 76 | -0.29 | 25 | 74 | 74 | 75 | 77 |
| Israel | TT | 90 | 0.49 | 37 | 69 | 100 | 95 | 90 | -0.18 | 36 | 65 | 88 | 89 | 92 |
| Italy | TT | 86 | 0.25 | 33 | 66 | 100 | 93 | 81 | -0.2 | 34 | 62 | 77 | 79 | 86 |
| Ivory Coast | TR | 66 | -0.5 | 45 | 49 | 45 | 56 | 79 | -0.23 | 51 | 45 | 65 | 72 | 64 |
| Jamaica | TR | 76 | -0.35 | 39 | 56 | 62 | 69 | 81 | -0.17 | 37 | 48 | 74 | 77 | 73 |
| Japan | BO | 93 | -0.01 | 28 | 71 | 100 | 96 | 82 | -0.27 | 30 | 65 | 77 | 79 | 88 |
| Jordan | TR | 92 | -0.24 | 36 | 68 | 87 | 89 | 90 | -0.41 | 44 | 65 | 72 | 81 | 85 |
| Kenya | TR | 84 | -0.15 | 46 | 56 | 78 | 81 | 82 | -0.35 | 43 | 52 | 65 | 74 | 77 |
| Kiribati | TR | 90 | -0.01 | 31 | 57 | 97 | 93 | 81 | -0.31 | 36 | 45 | 66 | 73 | 83 |
| Kuwait | TR | 94 | -0.01 | 35 | 61 | 100 | 97 | 91 | -0.38 | 39 | 51 | 72 | 81 | 89 |
| Latvia | TT | 74 | 0 | 25 | 69 | 85 | 79 | 86 | -0.14 | 34 | 64 | 86 | 86 | 83 |
| Lebanon | TT | 89 | -0.03 | 43 | 57 | 91 | 90 | 92 | -0.15 | 49 | 46 | 81 | 87 | 88 |
| Liberia | TR | 67 | -0.51 | 40 | 51 | 47 | 57 | 78 | -0.23 | 46 | 47 | 66 | 72 | 65 |
| Libya | TT | 100 | 0 | 37 | 47 | 100 | 100 | 83 | -0.2 | 44 | 39 | 70 | 76 | 88 |
| Lithuania | TT | 90 | -0.03 | 26 | 70 | 100 | 95 | 83 | -0.14 | 34 | 67 | 84 | 83 | 89 |
| Madagascar | TR | 88 | -0.27 | 37 | 56 | 77 | 83 | 80 | -0.35 | 40 | 52 | 65 | 73 | 78 |
| Malaysia | TR | 90 | -0.44 | 35 | 67 | 73 | 81 | 85 | -0.41 | 44 | 64 | 68 | 76 | 79 |
| Maldives | TR | 94 | -0.18 | 36 | 53 | 87 | 90 | 81 | -0.33 | 41 | 40 | 63 | 72 | 81 |
| Malta | TT | 89 | -0.09 | 23 | 72 | 97 | 93 | 80 | -0.2 | 28 | 68 | 80 | 80 | 86 |
| Marshall Islands | TR | 94 | 0 | 34 | 56 | 100 | 97 | 84 | -0.34 | 36 | 42 | 66 | 75 | 86 |
| Mauritania | TR | 94 | 0.02 | 38 | 51 | 100 | 97 | 78 | -0.17 | 46 | 47 | 69 | 74 | 85 |
| Mauritius | TR | 90 | -0.01 | 31 | 69 | 100 | 95 | 80 | -0.32 | 27 | 66 | 73 | 77 | 86 |
| Mexico | TR | 93 | -0.49 | 35 | 61 | 70 | 81 | 80 | -0.18 | 37 | 58 | 75 | 77 | 79 |
| Micronesia | TR | 95 | 0.24 | 30 | 58 | 100 | 98 | 81 | -0.31 | 34 | 44 | 67 | 74 | 86 |
| Monaco | TT |  |  |  |  |  |  | 85 | -0.17 | 28 | 62 | 85 | 85 | 43 |
| Morocco | TT | 86 | 0.02 | 39 | 61 | 94 | 90 | 74 | -0.26 | 46 | 60 | 65 | 70 | 80 |
| Mozambique | TR | 93 | -0.12 | 35 | 59 | 93 | 93 | 82 | -0.34 | 37 | 54 | 68 | 75 | 84 |
| Myanmar | TR | 93 | -0.13 | 52 | 39 | 81 | 87 | 84 | -0.38 | 58 | 32 | 55 | 70 | 78 |
| Namibia | TR | 95 | 0.02 | 25 | 64 | 100 | 97 | 78 | -0.2 | 32 | 61 | 75 | 76 | 87 |
| Nauru | TR | 98 | 0 | 30 | 58 | 100 | 99 | 79 | -0.33 | 36 | 44 | 64 | 71 | 85 |
| Netherlands | TT | 78 | 0.38 | 23 | 79 | 100 | 89 | 80 | -0.21 | 30 | 76 | 81 | 81 | 85 |
| Netherlands Caribbean Territories | TR | 97 | -0.19 | 27 | 69 | 98 | 97 | 82 | -0.18 | 29 | 64 | 81 | 82 | 90 |
| New Caledonia | TR | 81 | -0.36 | 37 | 51 | 65 | 73 | 83 | -0.34 | 39 | 43 | 65 | 74 | 74 |
| New Zealand | TR | 84 | -0.18 | 13 | 80 | 92 | 88 | 79 | -0.21 | 17 | 74 | 83 | 81 | 85 |
| Nicaragua | TR | 66 | -0.33 | 40 | 52 | 53 | 59 | 84 | -0.16 | 43 | 44 | 75 | 79 | 69 |
| Nigeria | TR | 67 | -0.01 | 54 | 44 | 64 | 65 | 78 | -0.23 | 65 | 37 | 59 | 68 | 67 |
| North Korea | TT | 99 | 0.05 | 47 | 44 | 100 | 100 | 80 | -0.2 | 55 | 38 | 65 | 72 | 86 |
| Norway | TT | 88 | -0.04 | 15 | 79 | 100 | 94 | 80 | -0.01 | 21 | 77 | 94 | 87 | 90 |
| Oman | TR | 97 | 0 | 28 | 60 | 100 | 98 | 82 | -0.29 | 31 | 52 | 72 | 77 | 88 |
| Pakistan | TR | 68 | -0.49 | 43 | 49 | 47 | 57 | 81 | -0.29 | 50 | 44 | 64 | 72 | 65 |
| Palau | TR | 96 | -0.01 | 27 | 64 | 100 | 98 | 82 | -0.32 | 31 | 59 | 72 | 77 | 88 |
| Panama | TR | 66 | -0.3 | 31 | 65 | 60 | 63 | 82 | -0.16 | 34 | 61 | 80 | 81 | 72 |
| Papua New Guinea | TR | 84 | -0.04 | 38 | 50 | 86 | 85 | 84 | -0.37 | 41 | 41 | 63 | 73 | 79 |
| Peru | TR | 74 | -0.49 | 34 | 56 | 55 | 64 | 79 | -0.22 | 39 | 52 | 71 | 75 | 70 |
| Philippines | TR | 86 | -0.48 | 40 | 57 | 63 | 75 | 84 | -0.38 | 51 | 52 | 63 | 73 | 74 |
| Poland | TT | 63 | 0 | 31 | 70 | 71 | 67 | 83 | -0.17 | 37 | 67 | 81 | 82 | 75 |
| Portugal | TT | 90 | 0.03 | 21 | 72 | 100 | 95 | 73 | -0.26 | 28 | 71 | 70 | 72 | 83 |
| Qatar | TR | 96 | 0 | 25 | 71 | 100 | 98 | 92 | -0.38 | 30 | 66 | 79 | 85 | 92 |
| Republic of the Congo | TR | 65 | -0.52 | 41 | 47 | 43 | 54 | 79 | -0.21 | 48 | 40 | 66 | 72 | 63 |
| Romania | TT | 100 | 0 | 28 | 62 | 100 | 100 | 82 | -0.24 | 31 | 57 | 76 | 79 | 89 |
| Russia | TT | 100 | 0 | 37 | 54 | 100 | 100 | 88 | 0.02 | 37 | 49 | 92 | 90 | 95 |
| Saint Kitts and Nevis | TR | 91 | 0 | 29 | 67 | 100 | 95 | 85 | -0.16 | 29 | 58 | 84 | 85 | 90 |
| Saint Lucia | TR | 66 | -0.02 | 32 | 72 | 73 | 70 | 84 | -0.16 | 29 | 68 | 86 | 85 | 77 |
| Saint Pierre and Miquelon | TT | 74 | -0.11 | 17 | 74 | 83 | 78 | 74 | -0.18 | 23 | 72 | 77 | 75 | 77 |
| Saint Vincent and the Grenadines | TR | 86 | -0.02 | 24 | 70 | 97 | 92 | 83 | -0.17 | 29 | 65 | 84 | 84 | 88 |
| Samoa | TR | 93 | 0.08 | 37 | 67 | 100 | 96 | 85 | -0.34 | 31 | 63 | 75 | 80 | 88 |
| Sao Tome and Principe | TR | 99 | -0.01 | 30 | 52 | 100 | 100 | 81 | -0.2 | 39 | 44 | 71 | 76 | 88 |
| Saudi Arabia | TR | 94 | -0.13 | 36 | 61 | 94 | 94 | 90 | -0.41 | 40 | 56 | 70 | 80 | 87 |
| Senegal | TR | 58 | -0.48 | 40 | 57 | 43 | 51 | 76 | -0.22 | 46 | 54 | 67 | 71 | 61 |
| Serbia and Montenegro | TT | 97 | -0.02 | 23 | 59 | 100 | 99 | 82 | -0.2 | 31 | 43 | 74 | 78 | 88 |
| Seychelles | TR | 95 | -0.05 | 28 | 64 | 100 | 98 | 81 | -0.35 | 33 | 62 | 69 | 75 | 86 |
| Sierra Leone | TR | 60 | -0.53 | 41 | 52 | 41 | 50 | 78 | -0.23 | 47 | 47 | 66 | 72 | 61 |
| Singapore | TR | 68 | -0.34 | 32 | 76 | 63 | 65 | 86 | -0.42 | 54 | 70 | 66 | 76 | 71 |
| Slovenia | TT | 97 | 0.01 | 29 | 72 | 100 | 99 | 84 | -0.19 | 45 | 68 | 80 | 82 | 90 |
| Solomon Islands | TR | 78 | -0.26 | 36 | 59 | 71 | 75 | 83 | -0.36 | 40 | 57 | 67 | 75 | 75 |
| Somalia | TR | 78 | -0.5 | 52 | 34 | 47 | 63 | 83 | -0.33 | 56 | 25 | 56 | 70 | 66 |
| South Africa | TT | 90 | -0.16 | 35 | 63 | 88 | 89 | 76 | -0.22 | 32 | 60 | 72 | 74 | 81 |
| South Korea | TT | 46 | -0.49 | 31 | 69 | 37 | 41 | 78 | -0.25 | 39 | 61 | 71 | 74 | 58 |
| Spain | TT | 69 | 0.01 | 30 | 70 | 79 | 74 | 76 | -0.24 | 31 | 67 | 73 | 74 | 74 |
| Sri Lanka | TR | 94 | 0.14 | 43 | 58 | 100 | 97 | 82 | -0.33 | 46 | 54 | 66 | 74 | 86 |
| Sudan | TR | 96 | 0 | 47 | 43 | 95 | 95 | 89 | -0.41 | 51 | 33 | 59 | 74 | 85 |
| Suriname | TR | 99 | -0.01 | 29 | 55 | 100 | 100 | 78 | -0.24 | 35 | 48 | 68 | 73 | 86 |
| Sweden | BO | 84 | 0.01 | 15 | 81 | 100 | 92 | 87 | -0.18 | 25 | 76 | 91 | 89 | 90 |
| Syria | TT | 95 | 0.02 | 44 | 53 | 99 | 97 | 92 | -0.14 | 53 | 46 | 82 | 87 | 92 |
| Taiwan | TR | 95 | -0.01 | 32 | 72 | 100 | 98 | 84 | -0.33 | 36 | 69 | 74 | 79 | 88 |
| Tanzania | TR | 81 | -0.42 | 36 | 58 | 65 | 73 | 82 | -0.36 | 41 | 53 | 66 | 74 | 73 |
| Thailand | TR | 92 | -0.42 | 38 | 61 | 73 | 82 | 87 | -0.38 | 46 | 57 | 68 | 78 | 80 |
| Togo | TR | 99 | 0.05 | 46 | 49 | 100 | 99 | 78 | -0.23 | 52 | 43 | 64 | 71 | 85 |
| Tonga | TR | 93 | -0.33 | 35 | 55 | 78 | 86 | 85 | -0.3 | 37 | 41 | 69 | 77 | 81 |
| Trinidad and Tobago | TR | 88 | -0.08 | 30 | 60 | 92 | 90 | 82 | -0.16 | 35 | 53 | 78 | 80 | 85 |
| Tunisia | TT | 94 | 0.05 | 34 | 61 | 100 | 97 | 82 | -0.22 | 42 | 57 | 73 | 77 | 87 |
| Turkey | TT | 86 | -0.03 | 36 | 62 | 91 | 89 | 81 | -0.19 | 43 | 59 | 75 | 78 | 83 |
| Tuvalu | TR | 100 | 0.03 | 29 | 59 | 100 | 100 | 82 | -0.35 | 33 | 45 | 66 | 74 | 87 |
| Ukraine | TT | 91 | 0 | 43 | 55 | 95 | 93 | 81 | -0.25 | 43 | 50 | 70 | 75 | 84 |
| United Arab Emirates | TR | 96 | 0.43 | 28 | 67 | 100 | 98 | 91 | -0.38 | 36 | 59 | 75 | 83 | 90 |
| United Kingdom | TT | 74 | 0.04 | 21 | 76 | 90 | 82 | 78 | -0.26 | 31 | 73 | 76 | 77 | 79 |
| United States | BO | 76 | -0.34 | 22 | 65 | 70 | 73 | 80 | -0.18 | 25 | 53 | 78 | 79 | 76 |
| Uruguay | TT | 92 | -0.01 | 25 | 66 | 100 | 96 | 74 | -0.23 | 28 | 63 | 71 | 72 | 84 |
| USA Caribbean Territories | TR | 89 | 0.23 | 30 | 59 | 100 | 95 | 82 | -0.16 | 31 | 48 | 78 | 80 | 87 |
| USA Pacific Inhabited Territories | TR | 93 | -0.05 | 27 | 57 | 99 | 96 | 82 | -0.28 | 29 | 44 | 71 | 76 | 86 |
| Vanuatu | TR | 91 | -0.42 | 28 | 63 | 76 | 84 | 84 | -0.35 | 32 | 56 | 72 | 78 | 81 |
| Venezuela | TR | 84 | -0.25 | 47 | 44 | 69 | 76 | 81 | -0.18 | 53 | 36 | 66 | 73 | 75 |
| Vietnam | TR | 86 | -0.34 | 43 | 57 | 70 | 78 | 84 | -0.39 | 51 | 52 | 63 | 73 | 76 |
| Western Sahara | TR | 99 | 0.05 | 38 | 39 | 100 | 99 | 74 | -0.25 | 44 | 21 | 56 | 65 | 82 |
| Yemen | TR | 92 | -0.07 | 45 | 45 | 88 | 90 | 84 | -0.34 | 49 | 36 | 61 | 73 | 81 |
