# Supplementary material for: Assessing Global Marine Biodiversity Status within a Coupled Socio-Ecological Perspective
Source: PLoS One. 2013 Apr 11;8(4):e60284. doi: 10.1371/journal.pone.0060284 (PMC3623975; doi:10.1371/journal.pone.0060284)
Supplement: Table S7 — Species status per region and globally, with status scores recalculated excluding each taxon (Jackknife analysis). The differences between the ‘all taxa included’ status score and the scores with each taxon excluded individually are also presented averaged across all countries (mean ±SD) and as a percent difference (i.e. divided by the all taxa status score × 100). Each column has the scores with a particular taxon excluded as follows: corals (Cor); hagfishes (Hag); mangroves (Man); marine mammals (Mar); other classes (Oth); reptiles (Rep); seagrasses (Sea); sharks, rays and skates (Sha); angelfish (fAn); butterflyfish (fBu); groupers (fGr); other fish (fOt); parrotfish (fPa); tunas & billfishes (fTu); wrasses (fWr). In order to compare taxonomic group effects by geographical area, the mean absolute difference (Diff) across excluded groups was obtained for each reporting region. (DOCX) [file pone.0060284.s015.docx]

| **EEZ** | **Status** | **Sha** | **Mar** | **Rep** | **fWr** | **Cor** | **Sea** | **fBu** | **fGr** | **Man** | **fAn** | **Hag** | **fOt** | **Oth** | **fTu** | **fPa** | **Diff** |
| --- | --- | --- | --- | --- | --- | --- | --- | --- | --- | --- | --- | --- | --- | --- | --- | --- | --- |
| **mean ±SD** |  | 2.05 ±1.69 | -1.65 ±2.39 | 0.97 ±0.76 | -0.93 ±1.04 | 0.32 ±1.34 | -0.25 ±0.39 | -0.17 ±0.18 | 0.14 ±0.35 | -0.11 ±0.30 | -0.10 ±0.12 | -0.07 ±0.16 | -0.03 ±2.35 | -0.04 ±0.12 | -0.02 ±0.84 | 0.02 ±0.03 |  |
| **percent ±SD** |  | 2.50 ±2.10 | -2.06 ±2.95 | 1.20 ±0.95 | -1.10 ±1.22 | 0.36 ±1.55 | -0.30 ±0.44 | -0.20 ±0.21 | 0.16 ±0.40 | -0.13 ±0.35 | -0.12 ±0.14 | -0.08 ±0.19 | -0.07 ±2.73 | -0.04 ±0.14 | -0.03 ±1.04 | 0.02 ±0.04 |  |
| **Global (area-weighted average)** | 82.7 | 83.87 | 81.28 | 83.54 | 82.29 | 83.15 | 82.61 | 82.58 | 82.7 | 82.67 | 82.64 | 82.67 | 81.95 | 82.69 | 82.94 | 82.71 | 0.63 |
| **Global (EEZ average)** | 83.1 | 85.17 | 81.46 | 84.09 | 82.18 | 83.43 | 82.86 | 82.95 | 83.25 | 83.01 | 83.02 | 83.04 | 83.08 | 83.08 | 83.09 | 83.13 | 0.78 |
| Albania | 85.1 | 90.41 | 84.61 | 85.68 | 81.18 | 84.47 | 84.24 | 85.07 | 85.72 | 85.07 | 85.07 | 84.87 | 85.07 | 85.07 | 85.11 | 85.07 | 0.99 |
| Algeria | 82.2 | 86.36 | 80.43 | 84.32 | 78.53 | 81.82 | 81.39 | 82.18 | 82.56 | 82.18 | 82.18 | 81.95 | 82.28 | 82.1 | 82.02 | 82.18 | 1.12 |
| Angola | 76.9 | 79.08 | 73.42 | 78.54 | 76.85 | 76.91 | 76.83 | 76.91 | 76.96 | 76.74 | 76.91 | 76.91 | 75.99 | 76.91 | 77.62 | 76.91 | 0.81 |
| Antarctica | 85.2 | 85.21 | 80.82 | 85.16 | 85.17 | 85.17 | 85.17 | 85.17 | 85.17 | 85.17 | 85.17 | 85.17 | 85.17 | 85.17 | 85.17 | 85.17 | 0.34 |
| Antigua and Barbuda | 82.4 | 85.54 | 80.77 | 83.06 | 82.12 | 82.17 | 82.33 | 82.31 | 82.56 | 82.35 | 82.32 | 82.42 | 81.56 | 82.42 | 81.43 | 82.45 | 0.68 |
| Argentina | 78.4 | 82.65 | 73.8 | 78.74 | 78.39 | 78.39 | 78.19 | 78.39 | 78.4 | 78.39 | 78.39 | 77.94 | 78.17 | 78.39 | 78.93 | 78.39 | 0.9 |
| Australia | 82.8 | 83.66 | 81.74 | 83.98 | 82.37 | 82.87 | 82.68 | 82.65 | 82.74 | 82.78 | 82.75 | 82.82 | 81.94 | 82.84 | 83.75 | 82.71 | 0.49 |
| Australian Southern Ocean Territories | 80.9 | 81.08 | 79.8 | 82.65 | 80.72 | 80.82 | 80.85 | 80.77 | 80.82 | 80.85 | 80.84 | 80.77 | 79.13 | 80.85 | 81.63 | 80.85 | 0.49 |
| Australian Tropical Territories | 81.3 | 81.45 | 75.95 | 82.02 | 81.24 | 81.41 | 81.34 | 81.3 | 81.32 | 81.34 | 81.32 | 81.34 | 80.37 | 81.34 | 82.7 | 81.35 | 0.72 |
| Bahamas | 84.1 | 86.35 | 83.06 | 84.88 | 83.35 | 83.5 | 83.93 | 83.83 | 84.62 | 83.96 | 83.83 | 84.1 | 83.98 | 84.11 | 83.68 | 84.2 | 0.6 |
| Bahrain | 90.7 | 91.34 | 90.85 | 90.17 | 90.05 | 93.69 | 90.51 | 90.45 | 90.73 | 90.64 | 90.51 | 90.69 | 90.69 | 90.69 | 90.69 | 90.69 | 0.42 |
| Bangladesh | 87.3 | 89.96 | 87.33 | 87.6 | 87.15 | 87.64 | 87.11 | 86.06 | 87.35 | 87.04 | 86.73 | 87.28 | 86.98 | 87.28 | 86.66 | 87.28 | 0.51 |
| Barbados | 80.4 | 83.91 | 77.38 | 82.15 | 80.09 | 80.09 | 80.28 | 80.26 | 80.62 | 80.32 | 80.26 | 80.36 | 79.32 | 80.36 | 78.98 | 80.39 | 0.99 |
| Belgium | 84.5 | 87.93 | 84.88 | 84.48 | 82.13 | 84.48 | 83.93 | 84.48 | 84.48 | 84.48 | 84.48 | 84.48 | 84.19 | 84.21 | 84.45 | 84.48 | 0.58 |
| Belize | 85.6 | 86.91 | 85.6 | 86.71 | 84.53 | 84.58 | 85.22 | 85.13 | 86.38 | 85.21 | 85.2 | 85.57 | 85.94 | 85.57 | 85.23 | 85.71 | 0.6 |
| Benin | 79.8 | 83.45 | 77.52 | 81.75 | 79.58 | 79.25 | 79.74 | 79.58 | 80.1 | 79.58 | 79.74 | 79.81 | 78.94 | 79.81 | 78.71 | 79.81 | 0.96 |
| Bosnia and Herzegovina | 84.6 | 91.15 | 84.05 | 85.12 | 81.21 | 84.2 | 84 | 84.58 | 85.12 | 84.58 | 84.58 | 84.39 | 83.71 | 84.58 | 84.36 | 84.58 | 1.09 |
| Brazil | 80.6 | 83.06 | 78.89 | 82.04 | 80.27 | 80.53 | 80.59 | 80.46 | 80.9 | 80.54 | 80.41 | 80.58 | 79.94 | 80.64 | 80.14 | 80.64 | 0.67 |
| British Caribbean Territories | 79.6 | 82.13 | 78.06 | 80.41 | 79.31 | 79.34 | 79.48 | 79.46 | 79.7 | 79.51 | 79.46 | 79.56 | 79.13 | 79.56 | 78.24 | 79.6 | 0.64 |
| British Indian Ocean Territory | 80.7 | 82.05 | 78.97 | 82.75 | 80.4 | 81.11 | 80.64 | 80.56 | 80.58 | 80.65 | 80.62 | 80.65 | 78.62 | 80.65 | 80.86 | 80.65 | 0.69 |
| British Pacific Territories (Pitcairn) | 83.0 | 84.01 | 83.48 | 82.99 | 82.65 | 83.28 | 82.99 | 82.86 | 83 | 82.99 | 82.94 | 82.99 | 80.34 | 82.99 | 84.7 | 82.99 | 0.54 |
| British Southern Ocean Territories | 79.2 | 80.05 | 70.86 | 79.7 | 79.17 | 79.21 | 79.18 | 79.19 | 79.19 | 79.21 | 79.2 | 79.14 | 78.67 | 79.21 | 80.36 | 79.21 | 0.97 |
| Bulgaria | 80.4 | 79.51 | 71.4 | 80.36 | 78.96 | 80.36 | 80.13 | 80.36 | 80.36 | 80.36 | 80.36 | 80.36 | 86.11 | 80.36 | 83.66 | 80.36 | 1.7 |
| Cambodia | 87.9 | 90.32 | 88.16 | 88.09 | 86.52 | 88.09 | 87.7 | 87.33 | 87.72 | 87.46 | 87.49 | 87.88 | 88.06 | 87.95 | 87.8 | 87.88 | 0.5 |
| Cameroon | 80.4 | 88.65 | 79.93 | 81 | 79.67 | 78.55 | 80.19 | 79.67 | 81.1 | 78.82 | 80.19 | 80.43 | 80.84 | 80.43 | 79.13 | 80.43 | 1.42 |
| Canada | 88.8 | 88.53 | 88.36 | 88.99 | 88.7 | 88.79 | 88.54 | 88.79 | 88.79 | 88.79 | 88.79 | 88.61 | 89.07 | 88.75 | 88.8 | 88.79 | 0.13 |
| Cape Verde | 77.1 | 79.77 | 73.48 | 78.69 | 76.89 | 76.69 | 76.98 | 76.94 | 77.26 | 77.07 | 77.03 | 77.07 | 76.74 | 77.07 | 76.86 | 77.07 | 0.82 |
| Chile | 78.7 | 80.56 | 76.97 | 79.82 | 78.63 | 78.67 | 78.69 | 78.67 | 78.66 | 78.68 | 78.68 | 78.6 | 77.08 | 78.68 | 78.33 | 78.68 | 0.58 |
| China | 82.0 | 86.42 | 81.47 | 82.58 | 81.18 | 81.51 | 81.99 | 81.37 | 81.67 | 81.81 | 81.85 | 81.98 | 81.53 | 81.93 | 81.26 | 81.97 | 0.75 |
| Clipperton Island | 80.3 | 82.79 | 76.81 | 83 | 80.28 | 80.06 | 80.27 | 80.25 | 80.23 | 80.27 | 80.3 | 80.27 | 77.94 | 80.27 | 80.2 | 80.27 | 0.95 |
| Colombia | 81.4 | 84.46 | 78.82 | 83.47 | 81.04 | 81.01 | 81.37 | 81.31 | 81.44 | 81.4 | 81.32 | 81.33 | 80.37 | 81.43 | 80.67 | 81.46 | 0.88 |
| Comoros | 82.2 | 83.99 | 80.38 | 83.66 | 81.67 | 83.63 | 82.16 | 82.02 | 82 | 82.19 | 82.15 | 82.23 | 80.92 | 82.25 | 82.69 | 82.26 | 0.77 |
| Costa Rica | 80.9 | 83.94 | 77.86 | 83.31 | 80.66 | 80.65 | 80.89 | 80.83 | 80.77 | 80.91 | 80.85 | 80.87 | 79.03 | 80.91 | 80.64 | 80.91 | 0.95 |
| Croatia | 84.6 | 89.84 | 83.93 | 85.4 | 80.97 | 84.08 | 83.78 | 84.6 | 85.23 | 84.6 | 84.6 | 84.45 | 84.48 | 84.6 | 84.52 | 84.6 | 1 |
| Cuba | 84.8 | 86.39 | 84.13 | 86.06 | 83.78 | 83.91 | 84.53 | 84.39 | 85.56 | 84.53 | 84.46 | 84.75 | 84.72 | 84.76 | 84.24 | 84.88 | 0.63 |
| Cyprus | 92.0 | 92.23 | 96.92 | 92.02 | 90.11 | 91.76 | 91.58 | 92.02 | 93.07 | 92.02 | 92.02 | 92.02 | 91.3 | 92.02 | 92.02 | 92.02 | 0.69 |
| Democratic Republic of the Congo | 86.3 | 87.61 | 91.64 | 86.27 | 85.85 | 86.27 | 85.85 | 86.27 | 87.23 | 82.93 | 86.27 | 86.27 | 86.32 | 86.27 | 85.65 | 86.27 | 0.97 |
| Denmark | 84.2 | 82.69 | 83.85 | 84.19 | 84.08 | 84.19 | 84.08 | 84.19 | 84.19 | 84.19 | 84.19 | 84.03 | 85.21 | 84.13 | 84.39 | 84.19 | 0.28 |
| Djibouti | 88.6 | 90.73 | 88.64 | 89.12 | 87.1 | 88.4 | 88.34 | 88.13 | 88.53 | 88.54 | 88.36 | 88.59 | 88.65 | 88.59 | 88.53 | 88.66 | 0.43 |
| Dominica | 82.6 | 85.55 | 80.62 | 84.24 | 82.02 | 82.25 | 82.43 | 82.39 | 82.89 | 82.43 | 82.4 | 82.61 | 82.05 | 82.61 | 81.56 | 82.68 | 0.83 |
| Dominican Republic | 83.0 | 85.69 | 81.11 | 84.25 | 82.54 | 82.66 | 82.91 | 82.85 | 83.37 | 82.93 | 82.87 | 83.03 | 82.45 | 83.04 | 82.15 | 83.1 | 0.73 |
| East Timor | 85.5 | 86.06 | 85.44 | 85.61 | 83.41 | 92.01 | 85.25 | 84.84 | 85.2 | 85.16 | 85.14 | 85.45 | 85.42 | 85.48 | 85.51 | 85.48 | 0.87 |
| Ecuador | 80.2 | 84.14 | 77.65 | 82.31 | 80.03 | 79.73 | 80.19 | 80.14 | 79.97 | 80.15 | 80.17 | 80.17 | 78.41 | 80.19 | 79.44 | 80.19 | 1.01 |
| Egypt | 90.7 | 91.22 | 94.03 | 90.74 | 88.94 | 92.74 | 90.36 | 90.62 | 91.28 | 90.7 | 90.65 | 90.71 | 90.26 | 90.71 | 90.72 | 90.74 | 0.68 |
| El Salvador | 81.8 | 85.01 | 79.46 | 84.15 | 81.49 | 81.57 | 81.78 | 81.76 | 81.41 | 81.81 | 81.78 | 81.62 | 80.34 | 81.86 | 81.48 | 81.84 | 0.92 |
| Equatorial Guinea | 78.7 | 82.54 | 75.41 | 80.41 | 78.42 | 78.18 | 78.62 | 78.48 | 78.77 | 78.5 | 78.62 | 78.68 | 77.76 | 78.68 | 78.55 | 78.68 | 0.96 |
| Eritrea | 88.7 | 89.83 | 88.62 | 89.15 | 87.02 | 90.88 | 88.42 | 88.27 | 88.6 | 88.62 | 88.45 | 88.66 | 88.7 | 88.66 | 88.6 | 88.73 | 0.5 |
| Estonia | 91.5 | 91.48 | 89.23 | 91.48 | 90.42 | 91.2 | 90.26 | 91.48 | 91.48 | 91.48 | 91.48 | 90.77 | 100 | 91 | 91.48 | 91.48 | 1.06 |
| Fiji | 83.3 | 84.75 | 82.71 | 84.2 | 82.83 | 84.49 | 83.22 | 83.1 | 83.17 | 83.24 | 83.17 | 83.25 | 81.83 | 83.26 | 83.13 | 83.26 | 0.53 |
| Finland | 95.8 | 95.75 | 94.09 | 95.75 | 95.52 | 95.66 | 95.07 | 95.75 | 95.75 | 95.75 | 95.75 | 95.28 | 100 | 95.37 | 95.75 | 95.75 | 0.54 |
| France | 80.0 | 82.91 | 78.33 | 81.09 | 78.1 | 79.88 | 79.54 | 79.99 | 80.31 | 79.99 | 79.99 | 79.74 | 79.94 | 79.79 | 80.08 | 79.99 | 0.75 |
| French Caribbean Territories | 82.2 | 85.31 | 80.08 | 83.62 | 81.67 | 81.84 | 82.01 | 81.98 | 82.4 | 82.05 | 82 | 82.17 | 81.51 | 82.17 | 81.06 | 82.23 | 0.83 |
| French Guiana | 79.4 | 82.18 | 76.83 | 81.31 | 79.32 | 79.37 | 79.37 | 79.02 | 79.78 | 79.09 | 78.84 | 79.24 | 79 | 79.37 | 78.11 | 79.37 | 0.89 |
| French Indian Ocean Territories | 81.9 | 84.02 | 80.12 | 82.77 | 81.66 | 82.56 | 81.9 | 81.83 | 81.87 | 81.93 | 81.89 | 81.94 | 80.19 | 81.95 | 82.84 | 81.94 | 0.7 |
| French Polynesia | 85.2 | 87.04 | 85.5 | 85.63 | 84.54 | 85.67 | 85.24 | 84.97 | 85.25 | 85.24 | 85.14 | 85.24 | 83.33 | 85.24 | 85.51 | 85.25 | 0.48 |
| French Southern Ocean Territories | 77.3 | 75.79 | 60.89 | 77.78 | 77.26 | 77.28 | 77.28 | 77.28 | 77.28 | 77.28 | 77.28 | 77.28 | 76.76 | 77.28 | 81.47 | 77.28 | 1.99 |
| Gabon | 77.5 | 81.49 | 73.93 | 79.02 | 77.33 | 76.95 | 77.38 | 77.22 | 77.67 | 77.16 | 77.39 | 77.46 | 76.82 | 77.46 | 77.36 | 77.46 | 0.98 |
| Gambia | 77.4 | 81.1 | 74.43 | 79.2 | 77.25 | 77.11 | 77.34 | 77.29 | 77.52 | 77.16 | 77.38 | 77.42 | 76.64 | 77.42 | 76.93 | 77.42 | 0.93 |
| Georgia | 84.3 | 84.32 | 76.28 | 84.31 | 80.5 | 84.31 | 83.6 | 84.31 | 84.31 | 84.31 | 84.31 | 84.31 | 90.99 | 84.31 | 87.46 | 84.31 | 1.77 |
| Germany | 83.4 | 85.84 | 82.77 | 83.38 | 81.4 | 83.36 | 82.29 | 83.38 | 83.38 | 83.38 | 83.38 | 83.34 | 86.33 | 82.76 | 83.68 | 83.38 | 0.81 |
| Ghana | 79.7 | 83.46 | 77.15 | 81.48 | 79.59 | 79.38 | 79.67 | 79.59 | 79.89 | 79.58 | 79.69 | 79.74 | 78.85 | 79.74 | 78.62 | 79.74 | 0.93 |
| Gibraltar | 79.8 | 85.87 | 79 | 82.2 | 76.37 | 79.43 | 79.05 | 79.79 | 79.97 | 79.79 | 79.79 | 79.61 | 79.13 | 79.61 | 79.21 | 79.79 | 1.3 |
| Greece | 88.0 | 89.56 | 88.68 | 88.25 | 85.45 | 87.66 | 87.35 | 88 | 88.67 | 88 | 88 | 87.99 | 87.37 | 87.98 | 88.7 | 88 | 0.61 |
| Grenada | 84.6 | 86.77 | 84.01 | 86.02 | 83.57 | 83.4 | 84.33 | 84.21 | 85.29 | 84.36 | 84.21 | 84.52 | 84.66 | 84.59 | 84.05 | 84.7 | 0.72 |
| Guatemala | 80.8 | 83.84 | 77.55 | 83 | 80.67 | 80.3 | 80.69 | 80.76 | 80.53 | 80.72 | 80.76 | 80.54 | 79.28 | 80.77 | 80.64 | 80.77 | 0.93 |
| Guinea | 77.3 | 81.85 | 75.1 | 79.34 | 77.01 | 76.62 | 77.22 | 77.01 | 77.44 | 77.02 | 77.19 | 77.28 | 76.73 | 77.28 | 76.13 | 77.28 | 1.06 |
| Guinea-Bissau | 77.4 | 81.45 | 74.89 | 79.33 | 77.18 | 76.85 | 77.34 | 77.18 | 77.55 | 77.08 | 77.33 | 77.41 | 76.72 | 77.41 | 76.78 | 77.41 | 0.98 |
| Guyana | 79.3 | 81.83 | 77 | 81.52 | 79.16 | 79.34 | 79.34 | 79.04 | 79.8 | 79.09 | 78.9 | 79.12 | 79.03 | 79.34 | 78.02 | 79.34 | 0.88 |
| Haiti | 84.8 | 86.58 | 83.79 | 86.1 | 83.86 | 84.07 | 84.55 | 84.43 | 85.54 | 84.54 | 84.49 | 84.77 | 84.53 | 84.77 | 84.18 | 84.89 | 0.67 |
| Honduras | 83.9 | 85.17 | 83.09 | 85.47 | 83.16 | 83.27 | 83.79 | 83.69 | 84.42 | 83.82 | 83.7 | 83.86 | 83.99 | 83.94 | 83.28 | 84.04 | 0.57 |
| Iceland | 75.7 | 71.02 | 76.49 | 75.73 | 75.73 | 75.7 | 75.41 | 75.73 | 75.73 | 75.73 | 75.73 | 75.6 | 78.33 | 75.51 | 76.34 | 75.73 | 0.83 |
| India | 82.9 | 85.87 | 80.33 | 84.83 | 82.59 | 83.18 | 82.81 | 82.56 | 82.85 | 82.75 | 82.75 | 82.92 | 81.76 | 82.92 | 82.71 | 82.92 | 0.83 |
| Indonesia | 84.0 | 84.91 | 83.31 | 84.63 | 82.77 | 88.04 | 83.87 | 83.56 | 83.82 | 83.8 | 83.78 | 84.01 | 83.6 | 84.03 | 84.23 | 84.03 | 0.75 |
| Iran | 88.1 | 89.9 | 87.71 | 88.31 | 87.49 | 89.01 | 87.87 | 87.75 | 88.13 | 88.04 | 87.92 | 88.08 | 87.89 | 88.08 | 87.97 | 88.08 | 0.38 |
| Iraq | 90.7 | 91.41 | 90.69 | 90.24 | 90.06 | 93.37 | 90.53 | 90.53 | 90.89 | 90.72 | 90.53 | 90.72 | 90.72 | 90.72 | 90.72 | 90.72 | 0.39 |
| Ireland | 77.6 | 77.42 | 75.46 | 78.24 | 77.1 | 77.55 | 77.51 | 77.61 | 77.61 | 77.61 | 77.61 | 77.35 | 78.63 | 77.36 | 78.15 | 77.61 | 0.49 |
| Israel | 91.2 | 92.21 | 95.61 | 91.22 | 88.85 | 90.92 | 90.73 | 91.22 | 92.64 | 91.22 | 91.22 | 91.22 | 90.43 | 91.22 | 91.22 | 91.22 | 0.79 |
| Italy | 82.7 | 87.59 | 79.26 | 83.53 | 79.9 | 82.36 | 81.92 | 82.7 | 83.11 | 82.7 | 82.7 | 82.51 | 83.01 | 82.69 | 83.07 | 82.7 | 1.16 |
| Ivory Coast | 79.9 | 83.67 | 77.45 | 81.61 | 79.71 | 79.42 | 79.91 | 79.71 | 80.16 | 79.67 | 79.84 | 79.91 | 79.01 | 79.91 | 78.87 | 79.91 | 0.94 |
| Jamaica | 82.1 | 83.86 | 80.1 | 84.05 | 81.71 | 81.82 | 82.02 | 81.96 | 82.33 | 82.04 | 81.98 | 82.1 | 81.58 | 82.1 | 81.17 | 82.15 | 0.69 |
| Japan | 83.0 | 85.18 | 82.2 | 83.82 | 82.56 | 83.37 | 82.93 | 82.77 | 82.93 | 82.98 | 82.88 | 82.98 | 81.61 | 82.98 | 82.88 | 82.98 | 0.52 |
| Jarvis Island | 81.0 | 84.37 | 79.42 | 82.67 | 80.55 | 80.96 | 80.96 | 80.82 | 80.91 | 80.96 | 80.9 | 80.96 | 78.77 | 80.97 | 80.21 | 81 | 0.85 |
| Jordan | 89.8 | 90.1 | 89.87 | 89.92 | 88.24 | 94.97 | 89.51 | 89.53 | 89.73 | 89.79 | 89.64 | 89.79 | 89.79 | 89.79 | 89.82 | 89.87 | 0.6 |
| Kenya | 82.8 | 85.63 | 81.38 | 84.31 | 82.16 | 83.46 | 82.74 | 82.63 | 82.69 | 82.79 | 82.73 | 82.83 | 81.58 | 82.84 | 82.31 | 82.85 | 0.76 |
| Kiribati | 81.8 | 83.76 | 80.15 | 83.22 | 81.44 | 82.28 | 81.78 | 81.66 | 81.73 | 81.78 | 81.72 | 81.78 | 79.98 | 81.78 | 81.86 | 81.8 | 0.65 |
| Kuwait | 90.5 | 91.35 | 90.47 | 90.08 | 89.79 | 92.54 | 90.28 | 90.25 | 90.57 | 90.47 | 90.28 | 90.47 | 90.47 | 90.47 | 90.47 | 90.47 | 0.35 |
| Latvia | 89.4 | 89.43 | 87.2 | 89.43 | 86.55 | 89.4 | 87.61 | 89.43 | 89.43 | 89.43 | 89.43 | 88.53 | 100 | 89.03 | 89.43 | 89.43 | 1.4 |
| Lebanon | 92.9 | 93.49 | 95.64 | 92.85 | 89.69 | 92.48 | 92.2 | 92.85 | 94.77 | 92.85 | 92.85 | 92.85 | 92.28 | 92.85 | 92.85 | 92.85 | 0.73 |
| Liberia | 79.7 | 83.24 | 76.87 | 81.47 | 79.54 | 79.35 | 79.67 | 79.5 | 79.83 | 79.52 | 79.63 | 79.67 | 78.71 | 79.67 | 78.67 | 79.67 | 0.93 |
| Libya | 84.7 | 86.23 | 79.83 | 85.63 | 82.79 | 84.48 | 84.29 | 84.7 | 85.22 | 84.7 | 84.7 | 84.7 | 85.65 | 84.7 | 85.37 | 84.7 | 0.94 |
| Lithuania | 86.8 | 86.79 | 84.66 | 86.79 | 83.31 | 86.79 | 84.66 | 86.79 | 86.79 | 86.79 | 86.79 | 85.8 | 100 | 86.08 | 86.79 | 86.79 | 1.74 |
| Madagascar | 81.7 | 83.88 | 79.79 | 82.92 | 81.1 | 82.4 | 81.59 | 81.48 | 81.49 | 81.63 | 81.59 | 81.66 | 80.28 | 81.69 | 82.14 | 81.66 | 0.74 |
| Malaysia | 84.6 | 85.76 | 84.49 | 84.99 | 83.44 | 88.13 | 84.49 | 84.11 | 84.43 | 84.42 | 84.35 | 84.62 | 84.59 | 84.65 | 84.51 | 84.64 | 0.62 |
| Maldives | 82.2 | 84.12 | 79.03 | 84.17 | 81.69 | 82.81 | 82.17 | 82.01 | 82.1 | 82.16 | 82.09 | 82.19 | 81.02 | 82.19 | 82.59 | 82.21 | 0.83 |
| Malta | 81.5 | 84.57 | 77.51 | 82.73 | 79.8 | 81.32 | 81.06 | 81.51 | 81.73 | 81.51 | 81.51 | 81.46 | 82.21 | 81.51 | 81.83 | 81.51 | 0.98 |
| Marshall Islands | 84.4 | 85.83 | 83.93 | 84.71 | 83.77 | 86.05 | 84.37 | 84.19 | 84.34 | 84.37 | 84.29 | 84.39 | 82.85 | 84.39 | 84.83 | 84.41 | 0.54 |
| Mauritania | 79.9 | 83.09 | 76.78 | 80.76 | 79.22 | 79.4 | 79.64 | 79.78 | 80.16 | 79.84 | 79.89 | 79.86 | 80.22 | 79.89 | 79.6 | 79.89 | 0.81 |
| Mauritius | 81.5 | 83.33 | 79.97 | 82.15 | 81.36 | 81.8 | 81.49 | 81.46 | 81.45 | 81.5 | 81.48 | 81.5 | 79.55 | 81.49 | 82.7 | 81.51 | 0.63 |
| Mexico | 80.9 | 84.22 | 78.56 | 82.89 | 80.64 | 80.65 | 80.82 | 80.81 | 80.75 | 80.89 | 80.85 | 80.87 | 79.68 | 80.94 | 80.36 | 80.94 | 0.88 |
| Micronesia | 82.2 | 83.16 | 80.56 | 83 | 81.91 | 83.12 | 82.18 | 82.06 | 82.14 | 82.19 | 82.14 | 82.21 | 81 | 82.21 | 82.98 | 82.22 | 0.56 |
| Monaco | 87.5 | 91.04 | 87.37 | 87.66 | 81.89 | 87.02 | 86.2 | 87.51 | 88.49 | 87.51 | 87.51 | 87.27 | 88.5 | 87.45 | 88.27 | 87.51 | 1.09 |
| Morocco | 76.4 | 78.34 | 74.94 | 78.21 | 75.41 | 76.26 | 76.26 | 76.35 | 76.42 | 76.35 | 76.35 | 76.34 | 76.15 | 76.34 | 75.55 | 76.35 | 0.66 |
| Mozambique | 82.7 | 84.94 | 80.95 | 84.05 | 81.88 | 83.01 | 82.6 | 82.43 | 82.52 | 82.61 | 82.56 | 82.67 | 81.6 | 82.72 | 82.93 | 82.69 | 0.69 |
| Myanmar | 84.2 | 86.17 | 81.6 | 85.77 | 83.88 | 84.89 | 84.12 | 83.84 | 84.12 | 84.07 | 84.06 | 84.21 | 83.57 | 84.22 | 83.95 | 84.22 | 0.7 |
| Namibia | 79.2 | 80 | 76.04 | 80.55 | 79.18 | 79.18 | 79.17 | 79.18 | 79.39 | 79.18 | 79.18 | 79.08 | 78.76 | 79.18 | 79.62 | 79.18 | 0.55 |
| Nauru | 80.2 | 80.95 | 77.88 | 81.88 | 79.98 | 80.67 | 80.16 | 80.09 | 80.12 | 80.15 | 80.12 | 80.16 | 78.96 | 80.16 | 80.67 | 80.16 | 0.61 |
| Netherlands | 82.8 | 86.18 | 82.69 | 82.82 | 80.93 | 82.82 | 82.41 | 82.82 | 82.82 | 82.82 | 82.82 | 82.82 | 82.74 | 82.35 | 82.85 | 82.82 | 0.51 |
| Netherlands Caribbean Territories | 83.2 | 85.95 | 81.62 | 84.52 | 82.47 | 82.64 | 83.02 | 82.92 | 83.48 | 82.98 | 82.91 | 83.05 | 82.88 | 83.17 | 82.34 | 83.25 | 0.75 |
| New Caledonia | 83.4 | 84.93 | 82.63 | 84.75 | 82.91 | 84.45 | 83.37 | 83.27 | 83.35 | 83.38 | 83.32 | 83.4 | 82.15 | 83.42 | 83.06 | 83.42 | 0.57 |
| New Zealand | 81.2 | 80.63 | 79.42 | 82.01 | 80.92 | 81.31 | 81.13 | 81.11 | 81.16 | 81.16 | 81.14 | 81.07 | 79.96 | 81.16 | 82.7 | 81.14 | 0.53 |
| Nicaragua | 84.6 | 86.61 | 83.97 | 86.04 | 83.66 | 83.9 | 84.38 | 84.29 | 84.55 | 84.43 | 84.32 | 84.46 | 84.37 | 84.57 | 84.13 | 84.63 | 0.58 |
| Nigeria | 79.3 | 82.97 | 77.02 | 81.31 | 79.04 | 78.67 | 79.21 | 79.04 | 79.52 | 79.01 | 79.21 | 79.29 | 78.65 | 79.29 | 78.34 | 79.29 | 0.96 |
| North Korea | 80.5 | 83.59 | 76.84 | 81.23 | 80.31 | 80.53 | 80.56 | 80.53 | 80.51 | 80.53 | 80.53 | 80.53 | 79.71 | 80.41 | 80.76 | 80.53 | 0.74 |
| Norway | 81.3 | 80.68 | 76.19 | 81.29 | 81.17 | 81.26 | 81.18 | 81.32 | 81.32 | 81.32 | 81.32 | 81.22 | 83.08 | 81.17 | 81.61 | 81.32 | 0.69 |
| Oman | 83.0 | 86.76 | 80.69 | 84.9 | 82.56 | 83.03 | 82.93 | 82.85 | 82.99 | 83.02 | 82.94 | 83.04 | 81.59 | 83.04 | 82.41 | 83.04 | 0.88 |
| Pakistan | 81.7 | 85.5 | 79.56 | 83.43 | 81.51 | 81.24 | 81.66 | 81.18 | 81.84 | 81.59 | 81.58 | 81.66 | 80.28 | 81.66 | 80.87 | 81.66 | 0.92 |
| Palau | 83.0 | 83.83 | 81.75 | 83.5 | 82.58 | 84.2 | 82.95 | 82.85 | 82.96 | 82.97 | 82.91 | 82.99 | 81.86 | 83 | 83.83 | 83 | 0.52 |
| Panama | 83.2 | 85.48 | 81.15 | 85.25 | 82.54 | 82.7 | 83.12 | 83.04 | 83.08 | 83.22 | 83.07 | 83.1 | 82.38 | 83.23 | 82.89 | 83.25 | 0.76 |
| Papua New Guinea | 84.3 | 84.7 | 83.49 | 85.45 | 83.42 | 87.04 | 84.25 | 84.04 | 84.19 | 84.21 | 84.16 | 84.33 | 83.61 | 84.35 | 84.59 | 84.35 | 0.61 |
| Peru | 80.5 | 85.64 | 77.87 | 82.29 | 80.22 | 80.52 | 80.52 | 80.42 | 80.11 | 80.51 | 80.51 | 80.37 | 78.85 | 80.52 | 79.57 | 80.52 | 1.09 |
| Philippines | 83.9 | 85.15 | 82.8 | 84.35 | 82.73 | 87.59 | 83.82 | 83.56 | 83.76 | 83.79 | 83.74 | 83.92 | 83.07 | 83.94 | 84.25 | 83.94 | 0.78 |
| Poland | 86.7 | 86.69 | 84.76 | 86.69 | 83.56 | 86.53 | 84.08 | 86.69 | 86.69 | 86.69 | 86.69 | 85.79 | 100 | 86.12 | 86.69 | 86.69 | 1.74 |
| Portugal | 74.8 | 74.93 | 73.57 | 76.54 | 74.27 | 74.75 | 74.76 | 74.8 | 74.97 | 74.8 | 74.8 | 74.79 | 74.65 | 74.77 | 74.16 | 74.8 | 0.42 |
| Qatar | 90.9 | 91.3 | 90.93 | 90.46 | 90.26 | 94.43 | 90.69 | 90.62 | 90.93 | 90.83 | 90.69 | 90.87 | 90.87 | 90.87 | 90.87 | 90.87 | 0.42 |
| Republic of the Congo | 80.9 | 84.04 | 78.26 | 81.42 | 80.66 | 80.86 | 80.66 | 80.52 | 81.1 | 80.33 | 80.86 | 80.86 | 80.72 | 80.86 | 80.34 | 80.86 | 0.7 |
| Romania | 84.7 | 83.76 | 81.45 | 84.7 | 82.91 | 84.7 | 84.05 | 84.7 | 84.7 | 84.7 | 84.7 | 84.7 | 89.55 | 84.7 | 85.95 | 84.7 | 1 |
| Russia | 88.7 | 87.83 | 94.61 | 88.74 | 88.7 | 88.73 | 88.68 | 88.73 | 88.73 | 88.73 | 88.73 | 88.73 | 88.01 | 88.66 | 88.8 | 88.73 | 0.58 |
| Saint Kitts and Nevis | 86.5 | 88.37 | 86.56 | 87.57 | 85.4 | 85.55 | 86.18 | 86.11 | 87.06 | 86.19 | 86.18 | 86.53 | 86.87 | 86.53 | 86.23 | 86.67 | 0.6 |
| Saint Lucia | 85.7 | 87.81 | 85.38 | 86.82 | 84.57 | 84.64 | 85.34 | 85.27 | 86.4 | 85.43 | 85.27 | 85.69 | 85.8 | 85.69 | 85.25 | 85.82 | 0.67 |
| Saint Pierre and Miquelon | 76.0 | 77.01 | 72.29 | 76.15 | 75.61 | 75.97 | 75.24 | 75.97 | 75.97 | 75.97 | 75.97 | 75.47 | 78.9 | 75.78 | 76.31 | 75.97 | 0.87 |
| Saint Vincent and the Grenadines | 84.7 | 86.59 | 83.76 | 86.12 | 83.88 | 83.98 | 84.41 | 84.36 | 85.24 | 84.41 | 84.36 | 84.64 | 84.4 | 84.65 | 83.98 | 84.75 | 0.66 |
| Samoa | 85.5 | 86.83 | 84.59 | 85.46 | 84.65 | 87.68 | 85.43 | 85.19 | 85.39 | 85.46 | 85.33 | 85.46 | 84.39 | 85.47 | 86.1 | 85.49 | 0.59 |
| Sao Tome and Principe | 82.0 | 85.84 | 80.16 | 84.06 | 81.32 | 80.99 | 81.9 | 81.61 | 81.8 | 81.78 | 81.9 | 82.03 | 81.39 | 82.03 | 81.78 | 82.03 | 0.94 |
| Saudi Arabia | 89.6 | 90.09 | 89.28 | 89.93 | 88.26 | 94.35 | 89.38 | 89.3 | 89.58 | 89.58 | 89.43 | 89.6 | 89.56 | 89.6 | 89.58 | 89.66 | 0.6 |
| Senegal | 77.7 | 81.13 | 74.5 | 79.37 | 77.42 | 77.23 | 77.59 | 77.52 | 77.94 | 77.5 | 77.68 | 77.71 | 77.31 | 77.71 | 77.37 | 77.71 | 0.91 |
| Serbia and Montenegro | 83.7 | 89.48 | 82.86 | 84.34 | 78.82 | 83.08 | 82.88 | 83.67 | 84.41 | 83.67 | 83.67 | 83.43 | 84.14 | 83.67 | 83.88 | 83.67 | 1.21 |
| Seychelles | 81.8 | 84.12 | 80.05 | 83.35 | 81.43 | 82.55 | 81.78 | 81.7 | 81.73 | 81.8 | 81.77 | 81.82 | 80.21 | 81.82 | 81.76 | 81.83 | 0.71 |
| Sierra Leone | 79.6 | 83.02 | 77.04 | 81.35 | 79.41 | 79.19 | 79.51 | 79.41 | 79.71 | 79.37 | 79.51 | 79.56 | 78.73 | 79.56 | 78.61 | 79.56 | 0.89 |
| Singapore | 84.9 | 85.33 | 85.03 | 84.62 | 83.81 | 93.1 | 84.68 | 84.39 | 84.7 | 84.44 | 84.65 | 84.91 | 84.92 | 84.91 | 84.84 | 84.91 | 0.93 |
| Slovenia | 85.5 | 89.28 | 85.03 | 86.73 | 82.26 | 85.08 | 84.68 | 85.49 | 86.1 | 85.49 | 85.49 | 85.49 | 84.96 | 85.49 | 85.37 | 85.49 | 0.87 |
| Solomon Islands | 83.8 | 84.07 | 83.03 | 84.84 | 83.21 | 85.97 | 83.76 | 83.62 | 83.71 | 83.76 | 83.69 | 83.82 | 82.76 | 83.83 | 84.32 | 83.83 | 0.55 |
| Somalia | 83.5 | 87.24 | 81.43 | 84.91 | 82.95 | 83.8 | 83.43 | 83.35 | 83.46 | 83.5 | 83.42 | 83.52 | 82.2 | 83.52 | 83.05 | 83.54 | 0.82 |
| South Africa | 78.8 | 79.26 | 69.16 | 79.9 | 78.66 | 78.65 | 78.74 | 78.71 | 78.83 | 78.77 | 78.75 | 78.7 | 77.88 | 78.78 | 81 | 78.78 | 1.26 |
| South Korea | 78.9 | 82.74 | 77.01 | 80.29 | 77.99 | 78.96 | 79.05 | 78.63 | 78.74 | 78.88 | 78.82 | 78.85 | 78.1 | 78.78 | 78.08 | 78.88 | 0.88 |
| Spain | 77.7 | 79.95 | 75.64 | 79.06 | 76.31 | 77.53 | 77.3 | 77.63 | 77.98 | 77.67 | 77.67 | 77.58 | 77.71 | 77.62 | 77.51 | 77.67 | 0.71 |
| Sri Lanka | 83.3 | 85.84 | 80.75 | 85.06 | 82.96 | 83.82 | 83.28 | 83.16 | 83.27 | 83.26 | 83.25 | 83.32 | 81.61 | 83.33 | 83.54 | 83.33 | 0.8 |
| Sudan | 88.1 | 89.22 | 86.7 | 89.02 | 86.82 | 91.54 | 87.89 | 87.78 | 88 | 88.05 | 87.93 | 88.08 | 87.95 | 88.08 | 88.04 | 88.14 | 0.69 |
| Suriname | 78.7 | 81.21 | 76.49 | 80.85 | 78.62 | 78.71 | 78.71 | 78.37 | 79.24 | 78.47 | 78.19 | 78.53 | 78.46 | 78.71 | 77.42 | 78.71 | 0.87 |
| Sweden | 90.0 | 90 | 88.33 | 89.95 | 87.99 | 89.84 | 88.76 | 89.95 | 89.95 | 89.95 | 89.95 | 89.32 | 98.79 | 89.34 | 90.02 | 89.95 | 1.12 |
| Syria | 93.7 | 94.2 | 95.34 | 93.73 | 90.96 | 93.37 | 92.97 | 93.73 | 95.52 | 93.73 | 93.73 | 93.73 | 93.11 | 93.73 | 93.73 | 93.73 | 0.6 |
| Taiwan | 84.1 | 86.31 | 83.45 | 85.26 | 83.03 | 85.15 | 83.96 | 83.65 | 83.84 | 84.02 | 83.87 | 84.07 | 83.32 | 84.1 | 83.8 | 84.08 | 0.65 |
| Tanzania | 82.6 | 84.97 | 81 | 84.12 | 81.94 | 83.32 | 82.53 | 82.39 | 82.43 | 82.55 | 82.5 | 82.6 | 81.31 | 82.63 | 82.45 | 82.62 | 0.72 |
| Thailand | 86.7 | 88.55 | 86.47 | 87.19 | 85.56 | 87.54 | 86.56 | 86.25 | 86.51 | 86.39 | 86.41 | 86.69 | 86.72 | 86.75 | 86.66 | 86.71 | 0.47 |
| Togo | 79.6 | 83.45 | 76.93 | 81.41 | 79.43 | 79.24 | 79.51 | 79.43 | 79.72 | 79.27 | 79.52 | 79.57 | 78.65 | 79.57 | 78.38 | 79.57 | 0.97 |
| Tonga | 85.8 | 87.38 | 85.91 | 86.24 | 85.02 | 86.79 | 85.74 | 85.54 | 85.73 | 85.74 | 85.65 | 85.76 | 84.27 | 85.76 | 85.57 | 85.79 | 0.48 |
| Trinidad and Tobago | 83.6 | 85.5 | 82.43 | 85.19 | 82.82 | 82.8 | 83.39 | 83.29 | 84.09 | 83.32 | 83.28 | 83.4 | 83.39 | 83.55 | 82.87 | 83.63 | 0.7 |
| Tunisia | 83.1 | 88.95 | 82.41 | 84.33 | 79.79 | 82.72 | 82.31 | 83.09 | 83.44 | 83.09 | 83.09 | 82.9 | 82.74 | 83.05 | 82.68 | 83.09 | 1.09 |
| Turkey | 84.0 | 84.22 | 78.51 | 84.01 | 80.86 | 83.86 | 83.32 | 83.97 | 84.34 | 83.97 | 83.97 | 83.97 | 88.23 | 83.96 | 86.58 | 83.97 | 1.34 |
| Tuvalu | 83.1 | 84.32 | 81.95 | 83.19 | 82.81 | 84.37 | 83.1 | 82.98 | 83.01 | 83.09 | 83.04 | 83.1 | 81.92 | 83.1 | 83.99 | 83.11 | 0.51 |
| Ukraine | 84.6 | 84.35 | 79.62 | 84.6 | 83.05 | 84.6 | 83.07 | 84.6 | 84.6 | 84.6 | 84.6 | 84.6 | 89.15 | 84.6 | 87.29 | 84.6 | 1.22 |
| United Arab Emirates | 90.1 | 91.23 | 90.04 | 89.7 | 89.47 | 92.02 | 89.88 | 89.81 | 90.14 | 90.01 | 89.89 | 90.07 | 90.09 | 90.07 | 90.03 | 90.07 | 0.37 |
| United Kingdom | 79.9 | 80.9 | 79.52 | 80.07 | 78.81 | 79.86 | 79.57 | 79.88 | 79.93 | 79.88 | 79.88 | 79.63 | 81 | 79.5 | 79.97 | 79.88 | 0.41 |
| United States | 81.1 | 81.67 | 84.3 | 81.68 | 80.88 | 81.05 | 80.81 | 80.98 | 81.15 | 81.09 | 81.04 | 81.05 | 80.29 | 81.02 | 80.93 | 81.1 | 0.51 |
| Uruguay | 76.0 | 78.55 | 69.77 | 77.2 | 75.96 | 75.96 | 75.71 | 75.96 | 76.13 | 75.96 | 75.95 | 75.59 | 75.49 | 75.96 | 77.42 | 75.96 | 1.12 |
| USA Caribbean Territories | 83.5 | 86.32 | 81.67 | 84.44 | 82.9 | 83 | 83.34 | 83.25 | 83.7 | 83.34 | 83.25 | 83.45 | 82.92 | 83.45 | 82.67 | 83.52 | 0.71 |
| USA Pacific Inhabited Territories | 83.1 | 85.27 | 81.94 | 83.5 | 82.77 | 83.88 | 83.08 | 82.97 | 83.07 | 83.09 | 83.02 | 83.08 | 81.13 | 83.09 | 83.55 | 83.11 | 0.6 |
| USA Pacific Uninhabited Territories | 83.3 | 85.94 | 83.04 | 83.88 | 83.02 | 83.36 | 83.33 | 83.21 | 83.31 | 83.33 | 83.3 | 83.33 | 81 | 83.33 | 83.26 | 83.35 | 0.51 |
| Vanuatu | 85.0 | 86.18 | 84.58 | 86.01 | 84.3 | 86.99 | 84.96 | 84.81 | 84.98 | 84.98 | 84.87 | 85.04 | 83.9 | 85.05 | 84.99 | 85.06 | 0.55 |
| Venezuela | 82.2 | 84.92 | 80.36 | 83.76 | 81.57 | 81.55 | 82.04 | 81.96 | 82.53 | 81.96 | 81.94 | 82.1 | 81.82 | 82.17 | 81.27 | 82.23 | 0.8 |
| Vietnam | 84.2 | 86.16 | 83.81 | 85.07 | 83.56 | 84.5 | 84.05 | 83.72 | 83.95 | 83.97 | 83.93 | 84.16 | 83.7 | 84.2 | 83.97 | 84.16 | 0.48 |
| Western Sahara | 76.5 | 78.8 | 73.16 | 78.22 | 75.93 | 76.4 | 76.38 | 76.43 | 76.51 | 76.45 | 76.45 | 76.35 | 76.55 | 76.45 | 75.96 | 76.45 | 0.77 |
| Yemen | 84.2 | 87.32 | 82.39 | 85.68 | 83.49 | 84.7 | 84.08 | 83.96 | 84.15 | 84.17 | 84.08 | 84.19 | 83.07 | 84.19 | 83.76 | 84.21 | 0.77 |
