# Supplementary material for: Assessing Global Marine Biodiversity Status within a Coupled Socio-Ecological Perspective
Source: PLoS One. 2013 Apr 11;8(4):e60284. doi: 10.1371/journal.pone.0060284 (PMC3623975; doi:10.1371/journal.pone.0060284)
Supplement: Table S8 — Pearson adjusted correlation coefficients (adjusted r2) of the linear regression of overall habitat score versus individual habitat scores. The correlations were obtained separately for reporting regions within three broad latitudinal ranges: tropical (TR, −30° to +30°), temperate and sub-tropical (TT, −30° to −60°, +30° to +60°) and boreal (BO, >60°, <−60°). The number of reporting regions is shown in parentheses. Correlations were excluded when the habitat occurred in less than 5 regions within that latitudinal range. Significance codes (p-values): <0.001 = ‘***’, <0.01 = ‘**’, <0.05 ‘*’, <0.1 = ‘.’, >0.1 = ‘ ’. See Table S6 for designation of latitudinal range. (DOCX) [file pone.0060284.s016.docx]

| **Lat** | **model** | **Parameter** | **estimate** | **Std. Error** | **t-value** | **P(>\|t\|)** | **Adj.r^2^** |
| --- | --- | --- | --- | --- | --- | --- | --- |
| TR | *corals* | Intercept | 0.66 | 0.05 | 14.32 | <0.001 | 0.17 (90) *** |
| TR |  | Slope | 0.23 | 0.05 | 4.33 | <0.001 |  |
| TR | *seagrasses* | Intercept | 0.58 | 0.04 | 15.9 | <0.001 | 0.38 (82) *** |
| TR |  | Slope | 0.31 | 0.04 | 7.14 | <0.001 |  |
| TR | *sea ice* | Intercept |  |  |  |  |  |
| TR |  | Slope |  |  |  |  |  |
| TR | *soft bottom* | Intercept | 0.52 | 0.11 | 4.64 | <0.001 | 0.06 (117) ** |
| TR |  | Slope | 0.33 | 0.12 | 2.83 | 0.006 |  |
| TR | *mangroves* | Intercept | 0.37 | 0.05 | 7.89 | <0.001 | 0.50 (84) *** |
| TR |  | Slope | 0.53 | 0.06 | 9.2 | <0.001 |  |
| TR | *salt marshes* | Intercept |  |  |  |  |  |
| TR |  | Slope |  |  |  |  |  |
| TT | *corals* | Intercept |  |  |  |  |  |
| TT |  | Slope |  |  |  |  |  |
| TT | *seagrasses* | Intercept | 0.55 | 0.11 | 4.85 | <0.001 | 0.32 (23) * |
| TT |  | Slope | 0.34 | 0.13 | 2.57 | 0.03 |  |
| TT | *sea ice* | Intercept | 0.72 | 0.05 | 13.59 | <0.001 | 0.40 (15) ** |
| TT |  | Slope | 0.22 | 0.07 | 3.22 | 0.007 |  |
| TT | *soft bottom* | Intercept | 0.21 | 0.26 | 0.8 | 0.43 | 0.11 (47) * |
| TT |  | Slope | 0.73 | 0.28 | 2.63 | 0.01 |  |
| TT | *mangroves* | Intercept |  |  |  |  |  |
| TT |  | Slope |  |  |  |  |  |
| TT | *salt marshes* | Intercept | 0.6 | 0.06 | 9.93 | <0.001 | 0.61 (12) ** |
| TT |  | Slope | 0.35 | 0.08 | 4.3 | <0.001 |  |
| BO | *corals* | Intercept |  |  |  |  |  |
| BO |  | Slope |  |  |  |  |  |
| BO | *seagrasses* | Intercept | 1.56 | 1.29 | 1.2 | 0.29 | 0 (6) |
| BO |  | Slope | -0.65 | 1.3 | -0.5 | 0.64 |  |
| BO | *sea ice* | Intercept | 0.54 | 0.17 | 3.29 | 0.01 | 0.32 (9) |
| BO |  | Slope | 0.41 | 0.19 | 2.18 | 0.07 |  |
| BO | *soft bottom* | Intercept | 0.49 | 0.52 | 0.93 | 0.38 | 0 (9) |
| BO |  | Slope | 0.44 | 0.56 | 0.79 | 0.46 |  |
| BO | *mangroves* | Intercept |  |  |  |  |  |
| BO |  | Slope |  |  |  |  |  |
| BO | *salt marshes* | Intercept |  |  |  |  |  |
| BO |  | Slope |  |  |  |  |  |
