# Supplementary material for: Assessing Global Marine Biodiversity Status within a Coupled Socio-Ecological Perspective
Source: PLoS One. 2013 Apr 11;8(4):e60284. doi: 10.1371/journal.pone.0060284 (PMC3623975; doi:10.1371/journal.pone.0060284)
Supplement: Text S1 — Supporting Methods and Results. (DOC) [file pone.0060284.s017.doc]

**Text S1. Supporting Methods and Results**

## Detailed gap-filling for habitat status and trends

All habitat scores were calculated by Exclusive Economic Zone (EEZ) region. Some habitat data were collected at a site-scale or regional scale within an EEZ. To calculate EEZ values for each habitat, we took the unweighted mean across all available values for a given habitat type. To calculate the single global score across all habitats, we calculated the mean across all EEZ scores weighted by their area.

For each habitat, we first assessed whether it was present or absent in a given region. If data were available for that region, no gap-filling was needed (i.e. ‘actuals’). If the habitat was present, but we had not data for it, it was gap-filled in the following order:

1. *Actuals*: as-is, no gap filling.
2. *Fitted*: linear prediction models calibrated with actuals. Note that these predictions are bounded to the 0-1 range.
3. *Adjacent*: neighboring regional averages, weighted by habitat area (extent).
4. *Georegion*: geographic regional averages, weighted by habitat area (extent).

Detailed methods for determining status and trends of habitats were originally reported in Halpern *et al.* [1].

### *Coral Reefs*

Coral reef extent data were derived from the 500m resolution dataset developed for Reefs at Risk Revisited [2]. We calculated extent using a resampled version of our EEZ regions to match their 500m resolution. For coral reefs, we measure condition from percent live coral cover reported from 12,634 surveys from 1975-2006 [3,4]. When multiple data points were available for the same site and year, the data were averaged to create one site estimate. We then averaged across all site data to calculate a per-country, per-year average. However, data were missing for several countries, and some countries did not have data for the reference or current year time periods; or had only 1-2 surveys. Because coral cover can be highly temporally and spatially dynamic, having only a few surveys that may have been motivated by different reasons (i.e. documenting a relatively pristine or an impacted habitat) can bias results. To calculate *C_k_* (Condition of habitat k) we created a fitted linear model of each country's data points from 1975-2010, provided that 2 or more points were in 1980-1995 and 2 or more points were in 2000-2010. We defined the current condition (health) as the mean of the predicted values for 2008-2010, and the reference condition as the mean of the predicted values for 1985-1987. Fitting a regression to estimate condition was more robust to data-poor situations and allowed us to take advantage of period(s) of intense sampling that did not always include both current and reference years. Where country data were not available, we used an average from adjacent EEZs weighted by habitat area, or a georegional average weighted by habitat area, based on countries within the same ocean basin, as described above and in Halpern *et al*. [1].

### *Mangroves*

Data on the extent of mangrove forests came from a global, raster-based, 30m resolution dataset [5]. To calculate condition of the habitat, we used FAO [6] data from 1980, 1990, 2000, and 2005 on a per country basis, with 2005 as the current condition, and 1980 as the reference condition. For trend in mangrove condition we used the rate of change in area over years 1980-2005. We used the reference condition data from 1980 as the basis for the habitat weights for pressures.

### *Seagrasses*

Seagrass extent was calculated from vector-based data from the Global Distribution of Seagrasses [7]. Seagrass status and trend data were calculated on a per-site, per-year basis using either estimated extent (km^2^) [8] or percent cover (%) [9]. The reference condition is the mean of the three earliest years from 1975-1985, or the two earliest years if needed. If data were not available, we fit a linear model to all data points, and then used the mean of the predicted values for 1979-1981 as the reference condition. For the current condition we used the mean of the three most recent years after 2000 or the two most recent years. If condition data satisfying these constraints were still not available, we fit a linear model to all data points, provided that there were at least three data points, and then used the mean of the predicted values for 2008-2010 as the current condition and the mean of the predicted values for 1979-1981 as the reference condition. Otherwise, we used neighboring (adjacent) regional averages, weighted by habitat area, or averages weighted by habitat area using seagrass geographical regions as defined by Hemminga and Duarte [10]. For trend, we used data after 2000 from Waycott *et al.* [8] or data from Short *et al.* [9] if there were at least 3 data points between 2001-2010. If these conditions were not met, we calculated trends from Waycott *et al.* [8] for the most recent 10 years after 1990 or else we used the mean of the trend in the adjacent regions or the regions within the corresponding seagrass geographical regions using the same methods described above for status.

### *Salt Marshes*

Salt marsh data per country came from multiple sources [11-15] and were generally reported as habitat area. We calculated annual change rates in habitat area using these habitat areas from 1994-2007, where available.  Most European data were obtained via the European Environment Agency databases housing information according to the European Union's Habitat Directive. Major data gaps exist for several key regions of the world, including the Middle East, South America, and Africa. The majority of the data included in the model come from North America (United States and Canada), Australia, New Zealand, China, Europe, and the United Kingdom. For trend estimates, we extracted categorical condition data (‘increasing”, “stable”, “declining” scored as 0.5, 0.0, and -0.5, respectively) from these sources on a per-country basis for countries where both a current and reference data year were available. For salt marsh trend we used the trend on these categorical values on a per-country basis.

### *Sea Ice*

Sea ice extent was calculated using sea ice concentrations from monthly 25 km (625km^2^ per pixel) resolution data from the National Snow and Ice Data Center [16]. Status was defined as the percentage of monthly pixels during a 3-yr smoothed average for 2009 (years 2008-2010) with ice concentrations of 15% or more, a standard concentration for defining the ocean-ice margin [17]. To calculate reference conditions, we used the long-term climatological mean across the whole time series of the data set (1979-2010). Using a long-term climatological mean dampened high frequency fluctuations and allowed for a more robust comparison of recent year values to the reference values. The trend calculation included the percent of monthly pixels per year with a 3-yr smoothed average from 2005 (years 2004-2006) to 2009 (years 2008-2010) with ice concentrations of 15% or more.

### *Subtidal soft-bottom*

Subtidal soft-bottom habitats within an EEZ were defined as the total area of shallow (0-60m) and shelf (60-200m) soft-bottom habitat within the reporting region based on benthic substrate point samples [18]. To estimate status of this habitat, we used intensity of trawl fishing as a proxy. Spatialized catch data are available from the global catch database of the *Sea Around Us* project [19]. The database was derived from FAO global fisheries catch statistics, data from international and national fisheries agencies, and reconstructed catch datasets [20]. The product of these sources of catch data was disaggregated spatially to a grid of 0.5° latitude by 0.5° longitude (259,200 grid cells globally) based on species distribution maps for over 1500 commercially exploited fish and invertebrate taxa and lists of fishing access agreements that regulate foreign access to the EEZs of maritime countries [21]. Catch data were available by gear type [21,22], and a subset of catch in tonnes from trawling and dredging gears was obtained by EEZ. We used the following gear types to define "trawled" catch: dredges, hand dredges, bottom trawls, and shrimp trawls, and we excluded mid-water trawls. We aggregated by summing the trawled catch data (1950-2006) by reporting region, and converted them to catch density by dividing annual catch by the trawlable (soft-bottom) area (i.e. based on the area of soft-bottom habitat within the EEZ). Because of extreme skew in these values, we log-transformed them and then rescaled them using the maximum density as the reference point. Status was then one minus the rescaled catch density in the most recent year (2006).

### *Pressures*

Pressures were calculated per EEZ region. In each EEZ region, we calculate the pressure based on the habitats present, weighted by their extent. A weight for a given pressure layer is the mean weight across habitats provided that at least one of the habitats present has a non-zero weight (Table S1).

**Species Sensitivity Analyses**

Species results were inherently biased by which taxa have been assessed by the IUCN Red List assessment process for extinction risk and have a described geographic distribution. Without a defined geographic species distribution we were not able to determine which species had ranges in particular Exclusive Economic Zones (EEZs), so non-spatial data were not included in our analyses. We used data on species extinction risks and distribution data available in early 2011 (Table S3). Since 2011, more species were assessed and geographically described, but we did not include them so that results presented here could more easily and directly be compared to previous results [1]. We conducted additional analyses to explore potential geographic and taxonomic biases in the data that may have affected our results or to highlight limitations of our approach.

### *Taxonomic Patterns*

All IUCN assessed species were from the animal and plant kingdoms. Mora *et al.* [23] counted and catalogued species for other broad kingdoms such as Chromista (n=4,859), Fungi (n=5,320), and Protozoa (n=8,118) beyond Animalia (n=171,082) and Plantae (n=8,600) for a total of 194,409 species catalogued. We used Bouchet’s [24] estimates for comparability at finer taxonomic resolution, and substituted more precise recent estimates for the smaller groups: marine mammals [25], and reptiles [26]. Based on species discovery rates, Mora *et al*. [23] predicted 2.21 million species in existence and the Census of Marine Life predicted that there are at least 1 million species [27]. Regardless of the exact numbers, assessed species (n=2,285) are orders of magnitude below the counts of those predicted [23,27], catalogued (n=229,715), and mapped (n=12,700). Comparing these species counts to all catalogued species at a coarser taxonomic level reveals large gaps in the state of our marine conservation biogeographic knowledge (Table S5, Fig. S1). Two notable exceptions were reptiles and marine mammals, both of which had relatively good coverage (> 50%).

To assess the taxonomic and spatial biases of any one of the 15 distinct taxonomic groups, we performed a jackknife analysis. Species were lumped into 15 taxonomic groups (Table S3) according to the groups that have been assessed as part of the IUCN Red List process, which typically convenes experts to conduct comprehensive species assessments by taxonomic group. Some taxa were represented to a finer taxonomic level (e.g. multiple families within the class Actinopterygii) while some groups were lumped across classes (e.g. corals include classes Anthozoa and Hydrozoa). Two “other” taxa were added to include taxa with distributions not already covered – other Actinopterygii and ‘all other taxonomic Classes’. By recalculating the species status score with any given group excluded we determined each group’s effect on the score. Some groups were more species rich than others, and some contained more endangered species (Table S3). Results of our jackknife analyses found that the greatest difference in the species status score across regions was only +2.5% (driven by sharks, rays and skates), with the average per-taxa absolute difference being less than mean ± 1 SD (Table S7; Figs. S5-S6)

### *Geographic Patterns*

To investigate geographic patterns and biases, we analyzed both the geographic patterns in the IUCN assessed species and species that have been mapped from a larger database of species distributions, Aquamaps [28]. A strong tropical bias for the number of species assessed is apparent in the Coral Triangle region (Fig. S2). In addition, many more species are described in coastal versus pelagic areas (Fig. S3). Of the total number of mapped species, far fewer have been assessed for extinction risk (Table S3). Areas Beyond National Jurisdiction are not well represented in terms of the percentage of species assessed, except for polar waters because of thorough assessments for some taxa such as marine mammals (Fig. S3).

Species extinction risk scores were lower in the poles (translating to higher status scores) because they were dominated by marine mammals, which typically had low extinction risk (Figure S4). Lower scores are most obvious in the North Atlantic where there are more sharks, tunas, and billfishes that tend to be more threatened.

For the jackknife analysis, we made one minor adjustment to our spatial allocation methodology for improved accuracy. Previously species scores in half degree cells were projected to Mollweide before performing a zonal summary and calculating the area-weighted average score per EEZ region. Here we instead intersected the EEZ regions in geographic space, calculated the cell area proportion within each region, and used a relational database query to calculate the area-weighted average. This provides a faster, more accurate measurement. This methodological change had only minor consequences for per-country scores (mean difference ± 1.3 ± 0.9%).

# References

1. Halpern BS, Longo C, Hardy D, McLeod KL, Samhouri JF, et al. (2012) An index to assess the health and benefits of the global ocean. Nature 488: 615-620.

2. Burke L, Reytar K, Spalding M, Perry AL, Cooper E, et al. (2011) Reefs at Risk Revisited. Washington, DC: World Resources Institute.

3. Bruno JF, Selig ER (2007) Regional Decline of Coral Cover in the Indo-Pacific: Timing, Extent, and Subregional Comparisons. Plos One 2.

4. Schutte VGW, Selig ER, Bruno JF (2010) Regional spatio-temporal trends in Caribbean coral reef benthic communities. Marine Ecology-Progress Series 402: 115-122.

5. Giri C, Ochieng E, Tieszen LL, Zhu Z, Singh A, et al. (2011) Status and distribution of mangrove forests of the world using earth observation satellite data. Global Ecology and Biogeography 20: 154-159.

6. FAO (2007) The world's mangroves 1980-2005. FAO Forestry Paper 153.

7. UNEP-WCMC (2005) Global Distribution of Seagrasses. In: UNEP-WCMC, editor. Cambrigdge.

8. Waycott M, Duarte CM, Carruthers TJB, Orth RJ, Dennison WC, et al. (2009) Accelerating loss of seagrasses across the globe threatens coastal ecosystems. Proceedings of the National Academy of Sciences of the United States of America 106: 12377-12381.

9. Short FT, Coles R, Fortes M, Koch E (2011) SeagrassNet. www.seagrassnet.org.

10. Hemminga MA, Duarte CM (2000) Seagrass Ecology. Cambridge: Cambridge University Press.

11. Bridgham SD, Megonigal JP, Keller JK, Bliss NB, Trettin C (2006) The carbon balance of North American wetlands. Wetlands 26: 889-916.

12. Dahl TE (2000) Status and trends of wetlands in the conterminous United States 1986 to 1997. Washington, D.C.: U.S. Department of the Interior, Fish and Wildlife Service. 82 p.

13. Ministry for the Environment (2007) Environment New Zealand 2007. Wellington, New Zealand: Ministry for the Environment.

14. JNCC (2004) (Joint Nature Conservation Committee). Common Standards Monitoring Guidance for Saltmarsh Habitats. ISSN 1743-8160.

15. EEA (2008) (Eionet, European Environment Agency). EU Habitats Directive Article 17 Reporting, 2001-2006.

16. Fetterer F, Knowles K, Meier W, Savoie M (2009) Sea Ice Index. In: Center' UNSaID, editor. Boulder, CO.

17. Johannessen OM, Bengtsson L, Miles MW, Kuzmina SI, Semenov VA, et al. (2004) Arctic climate change: observed and modelled temperature and sea-ice variability. Tellus Series a-Dynamic Meteorology and Oceanography 56: 328-341.

18. Halpern BS, Walbridge S, Selkoe KA, Kappel CV, Micheli F, et al. (2008) A global map of human impact on marine ecosystems. Science 319: 948-952.

19. Watson R, Kitchingman A, Gelchu A, Pauly D (2004) Mapping global fisheries: sharpening our focus. Fish and Fisheries 5: 168-177.

20. Zeller D, Pauly D (2007) Reconstruction of marine fisheries catches for key countries and regions (1950-2005). Vancouver, B.C. pp. 163 pp.

21. Watson R, Revenga C, Kura Y (2006) Fishing gear associated with global marine catches: II Trends in trawling and dredging. Fisheries Research 79: 103-111.

22. Watson R, Revenga C, Kura Y (2006) Fishing gear associated with global marine catches: I Database development. Fisheries Research 79: 97-102.

23. Mora C, Tittensor DP, Adl S, Simpson AGB, Worm B (2011) How Many Species Are There on Earth and in the Ocean? PLoS Biol 9: e1001127.

24. Bouchet P (2006) The magnitude of marine biodiversity. In: Duarte CM, editor. The exploration of marine biodiversity: scientific and technological challenges. Bilbao: Fundación BBVA. pp. 31-62.

25. Pompa S, Ehrlich PR, Ceballos G (2011) Global distribution and conservation of marine mammals. Proc Natl Acad Sci USA 108: 13600-13605.

26. Rasmussen AR, Murphy JC, Ompi M, Gibbons JW, Uetz P (2011) Marine Reptiles. PLoS ONE 6: e27373.

27. Ausubel JH, Crist DT, Waggoner PE (2010) First Census of Marine Life 2010. Washington, DC: Census of Marine Life.

28. Kaschner K, Rius-Barile J, Kesner-Reyes K, Garilao C, Kullander SO, et al. (2010) Predicted range maps for aquatic species. 8/30/2010 ed. www.aquamaps.org: Aquamaps.

29. Carpenter KE, Abrar M, Aeby G, Aronson RB, Banks S, et al. (2008) One-third of reef-building corals face elevated extinction risk from climate change and local impacts. Science 321: 560-563.

30. Polidoro BA, Carpenter KE, Collins L, Duke NC, Ellison AM, et al. (2010) The Loss of Species: Mangrove Extinction Risk and Geographic Areas of Global Concern. PLoS ONE 5: e10095.

31. Schipper J, Chanson JS, Chiozza F, Cox NA, Hoffmann M, et al. (2008) The status of the world's land and marine mammals: Diversity, threat, and knowledge. Science 322: 225-230.

32. Knapp L, Mincarone MM, Harwell H, Polidoro B, Sanciangco J, et al. (2011) Conservation status of the world's hagfish species and the loss of phylogenetic diversity and ecosystem function. Aquatic Conservation: Marine and Freshwater Ecosystems 21: 401-411.

33. Collette BB, Carpenter KE, Polidoro BA, Juan-Jordá MJ, Boustany A, et al. (2011) High Value and Long Life—Double Jeopardy for Tunas and Billfishes. Science 333: 291-292.

34. Sadovy de Mitcheson Y, Craig MT, Bertoncini AA, Carpenter KE, Cheung WWL, et al. (2012) Fishing groupers towards extinction: a global assessment of threats and extinction risks in a billion dollar fishery. Fish and Fisheries: no-no.
